# Supplementary material for: Restructuring personal networks with a Motivational Interviewing social network intervention to assist the transition out of homelessness: A randomized control pilot study
Source: PLoS One. 2022 Jan 21;17(1):e0262210. doi: 10.1371/journal.pone.0262210 (PMC8782388; doi:10.1371/journal.pone.0262210)
Supplement: S2 Appendix — This document includes exact text describing the RCT procedures approved by the author’s IRB prior to the trial beginning. The document includes both the original study plan, human subjects protection plan, and data safeguarding plan provided to the IRB in the initial ethics application as well as the final text uploaded into the human subjects review system which was discussed and approved in a full committee meeting prior to the trial starting. (PDF) [file pone.0262210.s002.pdf]

Study #:2013-0373

Last Update from Study Team: Mon Jan 11 14:32:16 PST 2016

1A Study Introduction

1A1 Study Description

1A1.1 ** **Title:**
Enhancing Housing First Programs with a Social Network Substance Use intervention

1A1.2 ** **Provide a brief summary or abstract for this study. Describes its purposes, study methods (i.e., participants, procedures, data sources), and expected results.** (5,000 character limit)

Substance abuse and HIV infection are interrelated public health problems facing the homeless. An estimated 30-50% of homeless adults are alcohol or drug dependent and homeless persons have been found to have rates of HIV infection 3 to 9 times greater than those with stable housing. Housing First (HF) programs attempt to address the health problems facing homeless persons by providing housing to those that have been chronically homeless without required abstinence from alcohol and other drug (AOD) use. Hundreds of HF programs have been established across the United States since 1990. These programs have demonstrated some success in reducing AOD use and sexual risk behavior in the short-term; however, the evidence demonstrating their effectiveness is limited. Approaches that are effective and easy to disseminate / implement are needed to enhance and prolong the positive behavioral effects of HF programs.
A number of recent studies from our research team and others suggest that the social networks of homeless individuals appear to influence their AOD use, recovery from co-occurring AOD and mental health disorders, engagement in HIV sexual risk behaviors, and even successful transition from homelessness. As such, targeting the social networks of HF residents may be a promising approach to enhance the positive behavioral outcomes associated with these programs. The social environments of homeless individuals are multi-dimensional in composition and structure: they include a mixture of risky and non-risky influences and sources of potential support and exhibit a range of structural features, from extremely dense to sparsely connected networks. As such, social network-based interventions should assist homeless individuals in making compositional changes to their network (adding and/or removing types of network members) as well as structural changes (strengthening, weakening, adding connections among network members) in order to have a positive impact on their AOD use and HIV sexual risk behaviors.
We propose a 3-year study to develop and pilot test a social network intervention for permanent supportive housing (PSH) program residents in the Skid Row area of Los Angeles. The intervention will be developed for residents at Skid Row Housing Trust (SRHT), one of the largest HF providers in Los Angeles that has an ongoing successful collaboration with members of our research team. SRHT case managers regularly conduct support meetings with residents during the first 6 months of residency. The proposed intervention will enhance these supportive interactions. Case managers will be trained to deliver the 30-minute intervention sessions using a motivational interviewing style.18 The motivational network intervention (MNI) will be conducted with the assistance of an electronic tablet and will involve five steps: (1) identifying members of a resident's personal social network and collecting information about network members (network composition); (2) asking the resident to rate the relationship strength of each unique dyad in their network in order to measure structural characteristics of the network; (3) displaying a network map that includes both structural and composition components of the resident's network; (4) discussing their social network's structure and composition in a non-judgmental and non-confrontational manner; and (5) exploring how the high-risk behavior in their social network impacts their own high-risk behavior and housing status. The MNI will be delivered across four sessions (baseline and two-week intervals).
Aim 1: To develop the MNI, train case managers to deliver the intervention, and test its feasibility and acceptability with 8-12 residents who have past-year harmful AOD use. We will iteratively beta-test an electronic MNI prototype based on feedback and refine/problem-solve recruitment/screening procedures prior to Aim 2.
Aim 2: To conduct a MNI pilot test with 60 residents with past-year harmful AOD use who are randomly assigned to receive the intervention or usual care. We will analyze baseline and post-intervention assessment data to determine the intervention's feasibility, acceptability and promise for a subsequent Stage 2 efficacy trial.19 We will explore the following hypotheses: intervention participants, compared to controls, will show: a) increased AOD and safe sex-related self-efficacy b) increased readiness to change AOD use and high-risk sexual behavior, c) social networks with fewer and less central high-risk members and d) significant reductions in AOD use and fewer HIV

sexual risk behaviors as compared to controls. We will also examine trends in the data to explore associations between other proximal outcomes (greater efficacy, greater readiness to change, reduced riskiness of networks) and the distal outcomes (changes in high-risk behavior).

1A1.3 **** Who is the intended audience for the results of this study? Where and in what form will you disseminate results? What are the potential benefits of the study to society?**

This study is significant because: (1) Homeless individuals are among the most vulnerable populations in the United States in need of health interventions, in particular interventions that address their alcohol and other drug use (AOD) and HIV risk; (2) Social network interventions are needed to improve HF programs, which provide permanent supportive housing (PSH) to homeless individuals without requiring alcohol and drug abstinence; (3) Existing social network interventions are few in number, complicated to implement, and do not address the structural complexity of homeless personal networks; (4) Providing HF case managers with technology to easily present social network information to their residents may enhance care; (5) Exploring network visualizations in a motivational interviewing style may enable HF residents to develop strategies for intervening in their own social networks; (6) Developing computerized interventions increases the potential that evidence based treatments reach broader populations and that treatments are delivered with fidelity.

1A1.4 **** Research Unit(s):**

- [ ] Arroyo Center
- [ ] Education and Labor
- [ ] Global Research Talent
- [x] **Health Care**
- [ ] HSOAC
- [ ] Justice, Infrastructure, and Environment *
- [ ] Labor and Population (Archive)
- [ ] National Security Research Division
- [ ] Project AIR FORCE
- [ ] Pardee RAND Graduate School
- [ ] RAND Europe
- [ ] Social and Economic Well-Being
- [ ] Other
- [ ] N/A

* JIE includes all work fomerly done under ISE, ICJ, and LBR.

1A2 Study Staff

1A2.1 **** Principal Investigator.** (If there are multiple PIs, please designate one PI here as being responsible for human subjects protection. Include the other PIs in the study staff list asked for in the next question.) The PI will receive all emails on the project from the HSPC. The PI in OASIS is the PI in RHINO.
David Kennedy

1A2.2 **Study Staff List.** Enter the names of all study staff whom you want to have view and edit privileges for the study file in RHINO. Note that these staff will receive all emails on the project from the HSPC.

Last Name	First Name	Organization
Hunter	Sarah	Behavioral and Policy Sciences

Last Name	First Name	Organization
Maksabedian	Ervant	PRGS Fellows
Osilla	Karen Chan	Behavioral and Policy Sciences
Tucker	Joan	Behavioral and Policy Sciences

1A2.3 **Primary HSPC Contact.** You can designate only one person as the primary contact. This can be the PI or a study staff member listed above or anyone else you choose. The Primary HSPC Contact should be someone who can be reached quickly if there is a question. The person designated a primary contact will receive all emails on the project from the HPSC.
David Kennedy

1A2.4 **Other Key Personnel.** In order to (1) determine the experience of the study staff and (2) avoid reviewer assignment conflicts, please identify other key personnel involved in the study. The individuals you list here will not receive email notifications or have view/edit rights on the study in RHINO. *Note: To avoid reviewer assignment conflicts, you can see if staff on your study would potentially be reviewers by looking at the HSPC membership list.*

Last Name	First Name	Organization	Role
There are no items to display			

1A2.5 **Email List.** Specify any other RAND staff (i.e., beyond the study staff) who should receive email notifications about this study. The persons you list here will have read-only access privileges to the study file.

Last Name	First Name	Organization	E-Mail
Chao	Catherine	Research Admin Operations	chao@rand.org
Golinelli	Daniela	Economics, Sociology and Statistics	daniela_golinelli@hotmail.com
Hunter	Sarah	Behavioral and Policy Sciences	shunter@rand.org
Osilla	Karen Chan	Behavioral and Policy Sciences	karenc@rand.org
Tucker	Joan	Behavioral and Policy Sciences	jtucker@rand.org

1A3 Screen for Involvement of Human Subjects and Eligibility for Exemption

1A3.1 ** **Before completing the full set of review questions, would you like to see if any of the population-procedure components of your study do not require review or are exempt from review?**

- ○ A. Yes - I want to see if all of the population-procedure components are exempt or do not require review.
- ◉ **B. Yes - I want to see if some of the population-procedure components are exempt or do not require review.**
- ○ C. No - Take me to the full set of the review questions.

1A3.3 You will be asked to list all of the components of your study and then select the components on which you will answer the screening questions. If these component(s) are determined to be exempt or do not require review, you will not need to answer the full set of review questions for those component(s). If you believe one of your components would not require review, list it separately.

Date: 1/13/2020, 10:22:21 AM
ID: 2013-0373

View: R2 - 1B Study Funding

Print Close

1B Study Funding

1B1 Funding Status

1B1.1 * **Funding Status:**

- ○ Proposed
- ○ Sponsor requires review before funding (Just in Time Review)
- ● **Funded**
- ○ Initiated, but not yet funded

1B2 * **Funding Sources.**
Click Add to list a funding source. To edit, click on the [Edit] link. To delete, check the checkbox next to the funding source to be removed, then click Delete.

	Name	Project Task Number	Proposal Number	Funding Institution Type	Instiution Other	Status
View	NIDA		HD234	Federal Govt		Completed

1B3 Prime Contractor

1B3.1 * **Which institution is the prime contractor or grantee?**
RAND

Version 2.15

VIEW4A0225673F000

5F Procedures - Benefits, Incentives, and Costs: Social Network Intervention

Related Populations:

- *Housing Project Residents (Full Committee - Approved)*

Show Tips for Navigating RHINO

5F2 Incentives and Other Forms of Compensation

5F2.1 * **Will the participants receive incentives or some other form of compensation (e.g., cash, voucher, meals, reimbursements)?**
yes

5F2.2 * **If "Yes", what will the participants receive as an incentive or other forms of compensation?**
Residents who agree to be participants in the intervention will be compensated for their time during the assessment interview sessions. Residents will be paid $30 for the baseline assessment interview, $40 for the post intervention assessment interview, and will be given a $5 gift card for each intervention session they attend.

5F2.3 * **If "Yes", how and when will the incentives or other forms of compensation be distributed to participants? Include any detail about conditions under which they might *not* receive the incentive or other form of compensation.**
Residents will be given gift cards for their participation in the baseline and post intervention assessment interviews and after each intervention session they attend.

5F3 Costs

5F3.1 * **Might the participants incur any financial costs that will not be reimbursed and that they would otherwise not incur?**
no

Version 2.10

* Required

OK Cancel

VIEW4A02378B88000

5G Procedures - Confidentiality Social Network Intervention

Related Populations:

- *Housing Project Residents (Full Committee - Approved)*

Show **Tips for Navigating RHINO**

5G1 Sensitive Information

5G1.1 * **Will any of the following types of sensitive information be intentionally accessed, acquired, or recorded as part of data collection about an individual? (Check all that apply)** *Note: You may have answered this screen 2H with regard to a specific population if you applied for an exemption for one or more population-procedure components of your study. However, we are asking this question again because the information may be sensitive with regard to another population in your study that you have associated with this procedure.*

☑	**A. Substance use**	**including alcohol, drugs, and tobacco**
☐	B. Physical health	including diagnosis, treatment, or any other private physical health information
☑	**C. Mental health**	**including diagnosis, treatment, current mental health status (e.g., suicidality), or any other private mental health information**
☐	D. Traumatic events	including victimization, bereavement, accidents, natural disasters, and military combat
☐	E. Illegal activity	including illicit drug use, human trafficking, and terrorism
☐	F. Immigration status	
☑	**G. Sexual behavior**	
☐	H. Abuse/neglect	including child, elder, domestic, and partner
☐	I. Educational records	including status, history, or performance
☐	J. Employment records	including status, history, or performance
☐	K. Financial information	
☐	L. Other	
☐	M. None of the above	

5G2 Identifying Information

5G2.1 * **Check all types of identifying information that your research will acquire about these participants.** *Note: You may have answered this question on screens 2B or 2C with regard to a specific population if you applied for an exemption for one or more population-procedure components of your study. However, we are asking this question again because the information may be identifiable with regard to another population in your study that you have associated with this procedure.*

☐	A. Names	including names appearing on consent forms, payment receipts, medical records, and emergency contact lists
☑	**B. Location and contacting information**	**including any address information smaller than state, telephone or fax numbers, email addresses, web URLs, and IP addresses**
☐	C. Dates	including birth date, marriage date, and treatment or visit dates from medical records
☐	D. Identification numbers	including Social Security Numbers, medical record or health plan beneficiary numbers, account numbers, student ID numbers, certificate or license numbers (e.g., driver's license), vehicle identifiers and serial numbers (e.g., license plates), and device identifiers and serial numbers (e.g., serial numbers on surgical implants)
☑	**E. Audio, video, or biometric indicators**	**including photographic or video images, audio recordings, and finger or voice prints**
☐	F. None of the identifiers listed above	by checking this box you certify that you are not obtaining any of the information listed above

5G3 Disclosure of Identifiable Person Level Data

5G3.1 * **Will any information that you collect go into person level non-research records (e.g., medical, educational, employment records) that are not maintained and controlled solely by the research team?** *Note: This is a relatively rare occurence for research done at RAND and would require HSPC approval.*
No

5G4 Reasons for Breaking Confidentiality

5G4.1 * **Could the procedure yield information potentially of importance to individual participants that they otherwise would be unlikely to know? This includes information specifically gathered or spontaneously provided or observed.**
○ Yes ◉ **No**

5G5 Promise of Confidentiality

5G5.1 * **Is confidentiality being promised to participants with no additional exceptions aside from those noted in 5G4?**
B. No, participants can choose to allow potentially identifiable information (e.g., quotes, performance information) to be included in published reports

5G5.5 * **Do you plan to apply for a Certificate of Confidentiality? Please note that such applications can be filed only after consent protocols have been approved.**
Yes

5G6 Identifiability by Inference in Published Reports

5G6.1 * **Might comments or other information included in published reports be identifiable by inference for participants who do not agree to be identified and are being assured of confidentiality?**
○ Yes ◉ **No**

5G7 Data Safeguarding Plan

5G7.1 * **Based on previous answers, a Data Safeguarding Plan (DSP) is required. Are you ready to upload it in draft or final form at this time?**
yes

5G7.2 You will be able to upload the Data Safeguarding Plan (DSP) later in Section 7

VIEW4A00CEE387800

5E Procedures - Risks:

Related Populations:

- *Housing Project Residents (Full Committee - Approved)*

In order to approve the study, the HSPC has to evaluate risk, determine that risks have been minimized, and determine that risks are resonable in relation to benefits.

Show **Tips for Navigating RHINO**

5E1 Breach of Confidentiality Risk

5E1.1 * **In the event a *breach of confidentiality allowing* someone outside of the research team to identify participants in this study, which of the following types of harm might result? (Check all that apply)**

A. Legal (i.e., criminal or civil liability)

C. Psychological (e.g., embarrassment, distress)

D. Social (e.g., damage to reputation)

5E2 Participation Risk

5E2.1 * **Aside from the risks associated with a breach of confidentiality, which of the following types of harm might *result from participation in the procedure?* (Check all that apply)**

C. Psychological consequences (e.g., distress, embarrassment, pressure to participate)

5E3 In the questions that follow, the phrase "individuals in the general U.S. population" refers to healthy individuals in the United States who are not incarcerated. Thus, the high standard of minimal risk is based on the amount of risk typically encountered in everyday life by healthy individuals in the United States. If the study population is unhealthy individuals, incarcerated individuals, or individuals in another country, the appropriate comparison is not how much risk "healthy individuals" would face. The comparison is what the actual subject population would face in comparison to miminal risk as defined by everyday life for healthy individuals in the United States. ***If a breach of confidentiality or participation would be dangerous in the study population, it is above minimal risk.***

5E4 Legal Risks Associated with a Breach of Confidentiality

5E4.1 * **Might the degree or amount of legal harm *resulting from a breach of confidentiality* be greater than ordinarily encountered in daily life by individuals in the general U.S. population?**
no

5E4.2 * **Might the likelihood of legal harm *resulting from a breach of confidentiality* be greater than ordinarily encountered in daily life by individuals in the**

general U.S. population?
no

5E4.3 * **Please explain the nature of the legal harm, what might cause it, and steps that would be taken to mitigate it.**
Participants may discuss participation in illegal activities which may expose them to legal risk. We will mitigate this risk by informing participants of the risks of disclosure and their option to not disclose any specific details that may put them at risk.

We will implement the following procedures to maintain data safeguarding and minimize the risk of a breach of confidentiality:

1. We will prepare and maintain a log of all sensitive data collected or acquired, including hardcopy and computer files. Date materials are received and returned or destroyed will be included.
2. We will train staff on data sensitivity and data safeguards being employed throughout the course of the study.
3. We will store and process sensitive hardcopies of documents in a centralized location with established access control procedures.
4. We will secure sensitive hardcopies of documents in locked files when not in use.
5. We will remove all names, addresses and other direct identifiers from hardcopies of documents and computer readable data. We will instead rely on codes for identification.
6. We will restrict access to shared disk files by restricting file permissions. Employing systematic monitoring procedures to insure that file permissions are correctly set for all files.
7. We will restrict access to locally stored disk files by utilizing encryption, password protection, or by storing the files on removable media kept in locked files when not in use.
8. We will print sensitive material, using a printer dedicated to the study, only when absolutely necessary. When it is necessary, we will make sure that only authorized personnel are at the printer when the sensitive material appears.
9. We will use certified mail, return receipt requested, for sensitive data and registered mail for very sensitive data when transferring materials by mail.
10. We will encrypt files containing sensitive information using a program, such as PGP, that provides RSA level security before sending them by email. The passwords for encrypted files will not be sent through e-mail, but may be transmitted through U.S. mail, commercial mail service, or directly to the intended recipient over the telephone (we will not leave passwords on voice mail systems or phone answering machines).
11. Any files containing sensitive information, including data files uploaded onto the RAND Accellion workspace, will be temporarily stored on local machines encrypted with whole disk PGP encryption software in addition to password protecting access to the computer.
12. We will create strong passwords to be used by research staff to access the Accellion workspace and these passwords will have no association with any other application or device.
13. All laptops used in baseline and follow-up interviews will be used exclusively for data collection and will have no new apps installed besides the EgoWeb app to minimize communication with the internet. Other than during data transmittal, the laptops will be disconnected from an internet connection.
14. We will re-train staff and review sensitive data inventory and data safeguards annually.
15. We will delete or modifying identifiers or data, which would allow individuals to be readily identified prior to the release of data outside the project or RAND.
16. We will destroy all individual linkages to data after the final report has been issued and reviewed unless specific plans for further longitudinal research have been proposed and approved by the person or group supplying the data.
17. For all participants who agree to participate in this study, space will be provided at the end of the consent form to share the names and contact information of two relatives or friends who may able to facilitate contact with them throughout this study. As with subject identifying information, any contact information collected will also be stored separately from the data files.
18. We will also ask that participants clarify their name(s) and the names of their contacts, given oftentimes, multiple names may be used. This information will be stored along with subject identifying information and will be used simply for the purpose of finding prospective participants if the need arises.
19. Staff members will report all serious violations of the Data Safeguarding Plan in writing to the Study Director and the Principal Investigator, with a copy to the Privacy Resource Office.
• All the above procedures will be implemented for each stage of the research process (e.g., sampling, data collection, data entry, etc.).

5E6 Psychological Risks Associated with a Breach of Confidentiality

5E6.1 * **Might the degree or amount of psychological harm *resulting from a breach of confidentiality* be greater than ordinarily encountered in daily life by individuals in the general U.S. population?**
○ Yes ◉ **No**

5E6.2 * **Might the <u>likelihood</u> of psychological harm *resulting from a breach of confidentiality* be greater than ordinarily encountered in daily life by individuals in the general U.S. population?**

( ) Yes (•) **No**

5E6.3 * **Please explain the nature of the discomfort or distress, what might cause it, and steps that would be taken to mitigate it.**

Participants will be discussing their behaviors, relationships, goals for changing their lives, and difficulties they experiencing transitioning from homelessness to residency at SRHT/SRO Housing. They may experience discomfort discussing these topics with case managers.

We will implement the following procedures to maintain data safeguarding and minimize the risk of a breach of confidentiality:

1. We will prepare and maintain a log of all sensitive data collected or acquired, including hardcopy and computer files. Date materials are received and returned or destroyed will be included.
2. We will train staff on data sensitivity and data safeguards being employed throughout the course of the study.
3. We will store and process sensitive hardcopies of documents in a centralized location with established access control procedures.
4. We will secure sensitive hardcopies of documents in locked files when not in use.
5. We will remove all names, addresses and other direct identifiers from hardcopies of documents and computer readable data. We will instead rely on codes for identification.
6. We will restrict access to shared disk files by restricting file permissions. Employing systematic monitoring procedures to insure that file permissions are correctly set for all files.
7. We will restrict access to locally stored disk files by utilizing encryption, password protection, or by storing the files on removable media kept in locked files when not in use.
8. We will print sensitive material, using a printer dedicated to the study, only when absolutely necessary. When it is necessary, we will make sure that only authorized personnel are at the printer when the sensitive material appears.
9. We will use certified mail, return receipt requested, for sensitive data and registered mail for very sensitive data when transferring materials by mail.
10. We will encrypt files containing sensitive information using a program, such as PGP, that provides RSA level security before sending them by email. The passwords for encrypted files will not be sent through e-mail, but may be transmitted through U.S. mail, commercial mail service, or directly to the intended recipient over the telephone (we will not leave passwords on voice mail systems or phone answering machines).
11. Any files containing sensitive information, including data files uploaded onto the RAND Accellion workspace, will be temporarily stored on local machines encrypted with whole disk PGP encryption software in addition to password protecting access to the computer.
12. We will create strong passwords to be used by research staff to access the Accellion workspace and these passwords will have no association with any other application or device.
13. All laptops used in baseline and follow-up interviews will be used exclusively for data collection and will have no new apps installed besides the EgoWeb app to minimize communication with the internet. Other than during data transmittal, the laptops will be disconnected from an internet connection.
14. We will re-train staff and review sensitive data inventory and data safeguards annually.
15. We will delete or modifying identifiers or data, which would allow individuals to be readily identified prior to the release of data outside the project or RAND.
16. We will destroy all individual linkages to data after the final report has been issued and reviewed unless specific plans for further longitudinal research have been proposed and approved by the person or group supplying the data.
17. For all participants who agree to participate in this study, space will be provided at the end of the consent form to share the names and contact information of two relatives or friends who may able to facilitate contact with them throughout this study. As with subject identifying information, any contact information collected will also be stored separately from the data files.
18. We will also ask that participants clarify their name(s) and the names of their contacts, given oftentimes, multiple names may be used. This information will be stored along with subject identifying information and will be used simply for the purpose of finding prospective participants if the need arises.
19. Staff members will report all serious violations of the Data Safeguarding Plan in writing to the Study Director and the Principal Investigator, with a copy to the Privacy Resource Office.

• All the above procedures will be implemented for each stage of the research process (e.g., sampling, data collection, data entry, etc.).

5E7 Social Risks Associated with a Breach of Confidentiality

5E7.1 * **Might the <u>degree or amount</u> of social harm *resulting from a breach of confidentiality* be greater than ordinarily encountered in daily life by individuals in the general U.S. population?**

( ) Yes (•) **No**

5E7.2 * **Might the <u>likelihood</u> of social harm *resulting from a breach of confidentiality* be greater than ordinarily encountered in daily life by individuals in the general U.S. population?**

○ Yes ◉ **No**

5E7.3 * **Please explain the nature of the social harm, what might cause it, and steps that would be taken to mitigate it.**

Participants will be discussing their behaviors, relationships, goals for changing their lives, and difficulties they experiencing transitioning from homelessness to residency at SRHT/SRO Housing. They may experience embarrassment or damage to their relationships with their social networks if discussions they have with case managers during the intervention becomes known to people in their social networks.

We will implement the following procedures to maintain data safeguarding and minimize the risk of a breach of confidentiality:

1. We will prepare and maintain a log of all sensitive data collected or acquired, including hardcopy and computer files. Date materials are received and returned or destroyed will be included.
2. We will train staff on data sensitivity and data safeguards being employed throughout the course of the study.
3. We will store and process sensitive hardcopies of documents in a centralized location with established access control procedures.
4. We will secure sensitive hardcopies of documents in locked files when not in use.
5. We will remove all names, addresses and other direct identifiers from hardcopies of documents and computer readable data. We will instead rely on codes for identification.
6. We will restrict access to shared disk files by restricting file permissions. Employing systematic monitoring procedures to insure that file permissions are correctly set for all files.
7. We will restrict access to locally stored disk files by utilizing encryption, password protection, or by storing the files on removable media kept in locked files when not in use.
8. We will print sensitive material, using a printer dedicated to the study, only when absolutely necessary. When it is necessary, we will make sure that only authorized personnel are at the printer when the sensitive material appears.
9. We will use certified mail, return receipt requested, for sensitive data and registered mail for very sensitive data when transferring materials by mail.
10. We will encrypt files containing sensitive information using a program, such as PGP, that provides RSA level security before sending them by email. The passwords for encrypted files will not be sent through e-mail, but may be transmitted through U.S. mail, commercial mail service, or directly to the intended recipient over the telephone (we will not leave passwords on voice mail systems or phone answering machines).
11. Any files containing sensitive information, including data files uploaded onto the RAND Accellion workspace, will be temporarily stored on local machines encrypted with whole disk PGP encryption software in addition to password protecting access to the computer.
12. We will create strong passwords to be used by research staff to access the Accellion workspace and these passwords will have no association with any other application or device.
13. All laptops used in baseline and follow-up interviews will be used exclusively for data collection and will have no new apps installed besides the EgoWeb app to minimize communication with the internet. Other than during data transmittal, the laptops will be disconnected from an internet connection.14. We will re-train staff and review sensitive data inventory and data safeguards annually.
15. We will delete or modifying identifiers or data, which would allow individuals to be readily identified prior to the release of data outside the project or RAND.
16. We will destroy all individual linkages to data after the final report has been issued and reviewed unless specific plans for further longitudinal research have been proposed and approved by the person or group supplying the data.
17. For all participants who agree to participate in this study, space will be provided at the end of the consent form to share the names and contact information of two relatives or friends who may able to facilitate contact with them throughout this study. As with subject identifying information, any contact information collected will also be stored separately from the data files.
18. We will also ask that participants clarify their name(s) and the names of their contacts, given oftentimes, multiple names may be used. This information will be stored along with subject identifying information and will be used simply for the purpose of finding prospective participants if the need arises.
19. Staff members will report all serious violations of the Data Safeguarding Plan in writing to the Study Director and the Principal Investigator, with a copy to the Privacy Resource Office.

• All the above procedures will be implemented for each stage of the research process (e.g., sampling, data collection, data entry, etc.).

5E11 Psychological Risks Resulting from Participation

5E11.1 * **Might the <u>degree or amount</u> of psychological harm resulting *from participation in the procedure itself* (i.e., other than from a breach of confidentiality) be greater than ordinarily encountered in daily life by individuals in the general U.S. population?**

○ Yes ◉ **No**

5E11.2 * **Might the <u>likelihood</u> of psychological harm resulting *from participation in the procedure itself* (i.e., other than from a breach of confidentiality) be greater than ordinarily encountered in daily life by individuals in the general U.S. population?**

○ Yes ◉ **No**

5E11.3 * **Please explain the nature of the psychological harm, what might cause it, and steps that would be taken to mitigate it.**

Participants will be discussing their behaviors, relationships, goals for changing their lives, and difficulties they experiencing transitioning from homelessness to residency at SRHT/SRO Housing. They may experience embarrassment and discomfort discussing these topics. They may also experience stress and/or discomfort attempting to implement their strategies for making changes in their lives to achieve the goals they set with their case managers.

To mitigate psychological harm, case managers will be trained to conduct interviews using motivational interviewing (MI) style. The MI training will follow the eight stages in learning MI including building motivation to change and strengthening commitment to change. We will focus on foundational elements of MI and the spirit or counseling style that underlies its techniques. The training will involve a mix of didactic presentation, demonstration, and several practice sessions involving role playing exercises that are geared toward people with little or no exposure to MI. Through this training, they will learn: key principles of MI, an empathic, yet directive, counseling style, semi-structured techniques to help people talk about change, how to "roll with resistance" with reflections, how to use information and feedback as a motivational tool, how to reinforce change talk, and how to help others commit to a change plan appropriate to their stage of change. During the second day of training, the case managers will become familiar with the 4-session protocol and successfully complete a “mock” interview. We recognize that training is an ongoing process and follow-up booster sessions will continue after this initial training. For example, Dr. Osilla will take notes during each practice session and will provide supervision to the case managers on their adherence to the protocol and their use of MI techniques throughout the intervention delivery during the pilot. She will listen to every audio recording and provide ongoing weekly supervision and feedback/coaching to intervention case managers via phone to promote MI behaviors, to sustain MI proficiency, and to enhance intervention fidelity.

If psychological reaction to interviews is greater than mild discomfort, we will implement an adverse event protocol in which participants will be referred to on-call clinicians through SRHT/SRO Housing or through RAND (Dr. Osilla). We will report adverse events to the RAND Human Subjects Protection Committee and evaluate procedures to determine of changes are necessary to avoid future adverse events.

Version 2.10

---

* Required

OK Cancel

VIEW49FE19647C800

5A - Procedures - Interventions: Overview: Social Network Intervention

Related Populations:

- *Housing Project Residents (Full Committee - Approved)*

You previously indicated that your research involved the following intervention procedure(s). If you wish to change your answer, please return to 3C1.1.
K. Socio-behavioral or educational intervention

Show **Tips for Navigating RHINO**

5A1 * **Provide a step-by-step description of this procedure for the studied groups of participants. Include both alterations of their experiences (e.g., novel educational practices, new health services) and administration of any treatments they might not otherwise receive (e.g., medications, nutritional supplements).** *Note: you will be asked below in 5A2 about assignment of participants to different groups (e.g., different data collection or treatment/control groups).*
The intervention will incorporate motivational interviewing and social network visualization. This Motivational Network Intervention (MNI) will consist of four sessions that build on each other.

The first MNI session will occur within two weeks after the resident completes the baseline interview. The MNI sessions will occur as a supplement to typical case management meetings and will be conducted by a RAND facilitator trained in motivational interviewing and the use of the MNI technology. The MNI part of this meeting will last approximately 30-45 minutes and will consist of two parts: a network interview with closed-ended network questions that cover the time period since their last interview and a discussion of a resulting network visualization conducted in a MI style.

The network interviews are a shortened version of interviews that RAND researchers conducted with homeless men and women, many of whom had low education levels (25-33% without high school education) and do not require respondents to be literate.

In the first session, case managers will read network questions from the MNI electronic interface verbatim. These structured network interview questions are similar to the questions asked at baseline but will primarily focus on characteristics to be displayed in network visualizations. The interviews will cover the timeframe between the baseline and first session (or between visits in subsequent sessions), and will consist of a network name generator to generate lists of network members (also known as "alters") (e.g., "List 10-15 people you have interacted with in the past 2 weeks."), network composition (e.g., "How likely do you think it is that these people will drink alcohol or use some type of drugs to get high in the next two weeks?" "Who have you ever had sex with?"), and network structure questions (e.g., "Does (Alter 1) know (Alter 2)?").

Answers to these questions provide raw data to generate network visualizations. To identify the key recent social experiences in their lives, participants will be asked to name 10-15 network ties who they have interacted with in the past two weeks and who have been significant in their lives, either positively or negatively. Participants will define significance for themselves but case managers will suggest that they consider those who have had an important emotional, social, financial, or other influential impact. Once case managers have elicited this list of 10-15 alters, they will confirm if any alters were named in the baseline assessment network interviews.

The MNI will be administered on RAND encrypted laptops. Files created by EgoWeb installed on these laptops will be uploaded to a RAND Accellion file uploading

site for this project. These files will be imported into a version of EgoWeb on RAND's extranet (egoweb.rand.org). For all alters named, case managers will ask participants a series of questions about the resident's perception of the behavior of the alter and their relationship with the alter.

For example, questions include how likely the resident thinks the alter will drink alcohol or use drugs to get high in the next two weeks, if the resident tends to drink or do more drugs than usual when they are with the alter, if they have had sex with the alter, if they used a condom with the alters who were sex partners, the respondents perception of the likelihood that the alter engages in risky sex, and if the alter provides the resident with any support (emotional support, encouragement, advice). After these questions about the alters, residents will be asked if each alter knows the other alters and how often they interact. Based on our previous network interviews and tests of the current MNI interface, we estimate that this process will take about 15 minutes to complete.
Once all network questions have been asked and answered, case managers will lead a discussion about the participant's network in a motivational interviewing (MI) style guided by the network visualizations. They will show the participant a series of 4 network diagrams customized for the participant. The initial diagram will present the network structure and highlight the alters who interact the most with the other members of the network. Subsequent diagrams will highlight drinking and drug use of the alters and the respondent with the alters, sex partners and the perception of the risky sexual behavior of the alters, and supportive alters. Case managers will explore the pros and cons of residents' current social network composition and structure and discuss their readiness, willingness and confidence to change things about their networks that they do not like, such as spending more or less time with certain network members or introducing network members to each other. Discussions will especially focus on relationships with sex partners and alters who either discourage or foster engagement in high-risk behaviors that lead to undesirable outcomes. Case managers will ask participants about their behaviors with these alters (safe sex decisions, AOD use), how their relationships with these alters impacted decisions about doing things they know put them at risk either currently or potentially in the future, and what they may want to change about their behaviors and relationships. They will also look for opportunities to encourage discussion of strategies/tactics for change that may take place or begin to take place over the upcoming two week interval and to set goals for themselves. For example, if the participant has not had any positive relationships, a case manager can ask the resident, "Is there someone you haven't named who you would like to interact with more? What are some steps you can take to interact with ____ in the next two weeks?").

For sessions 2 through 4, facilitators will print out network diagrams from previous interviews before interviews and give these to the resident. In addition to depicting the network, the printouts will have facilitator notes about the statements the resident made regarding their goals and the steps they are willing to take towards modifying their social network relationships over the next two weeks and will have space for residents to write their own notes.

The three subsequent sessions will also cover the time periods between sessions and will repeat the network interview and discussion. Discussions of the visualizations will focus on the ways in which the network changed between sessions, including discussing alters they either started or stopped interacting with since the previous MNI session and discussing what has not changed since the last interview. Case managers will review notes from previous interviews and will ask follow-up questions about attempts at change and encourage steps that residents are taking toward meeting their goals. The diagrams shown to residents will enable participants to continue contemplating how their goals for change have evolved. The in-depth discussions of the social environment of the resident and the documentation of the network composition/structure and stated goals about change will enhance the supportive relationship between the case manager and resident beyond the individual MNI sessions. The network visualizations/printouts resulting from the network interviews will provide both case managers and residents with a tangible output on which to focus discussions and make abstract discussions of social life, relationships, and social change more concrete. They will also provide documentation of the personal goals and aspirations of the residents across sessions and facilitate discussions of successful attempts at change to allow for reinforcement during MNI sessions and other interactions.

5A2 Administered to All Members

5A2.1 * **Will any subgroups of the population(s) referenced above receive different interventions? (Check all that apply)**

B. Yes - some participants will receive the socio-behavioral or clinical intervention and others will be in the control group

5A2.2 You selected "C" above in addition to "A" or "B". Please correct this before proceeding.

5A2.3 * **If "A", please describe how individuals will be selected for physical exams or the collection of biologic samples/specimens (e.g., random assignment of individuals/groups or based on some individual characteristics). If already described above in 5A1, please indicate that below.**

5A2.4 * **If "B", please describe how individuals will be selected into the intervention/treatment and control groups (e.g., random assignment of individuals/groups or quasi-experimental designs comparing existing groups). If already described above in 5A1, please indicate that below.**

Residents will be randomly assigned to usual case manager support or case manager support + MNI after their baseline interview using a stratified permuted block

randomization strategy. RAND will be responsible for this step. Although we will randomize the participants to one of two groups, given the smaller sample size, the randomization might produce an unbalanced assignment with respect to important characteristics. For this reason the randomization will be stratified with respect to gender and by AOD dependence status. About 26% of SRHT clients are women and about 32% are AOD dependent and stratifying these characteristics will ensure an approximately equal number of women and residents with AOD dependence assigned to both conditions. If the intervention and control groups turn out to be unbalanced with respect to other characteristics, we will use covariate adjustment at the analysis phase. The randomization sequence will be generated by Dr. Miles and kept hidden from the other team members so that the assignment to the two conditions cannot be gamed.

5A3 *** Describe the qualifications and/or training for those who will conduct the intervention.**
Training is consistent with our previous studies and will include a 2-day workshop that will cover two areas: Motivational Interviewing (MI) and the social networking interview protocol. The MI training will follow the eight stages in learning MI including building motivation to change and strengthening commitment to change. We will focus on foundational elements of MI and the spirit or counseling style that underlies its techniques. The training will involve a mix of didactic presentation, demonstration, and several practice sessions involving role playing exercises that are geared toward people with little or no exposure to MI. Through this training, they will learn: key principles of MI, an empathic, yet directive, counseling style, semi-structured techniques to help people talk about change, how to "roll with resistance" with reflections, how to use information and feedback as a motivational tool, how to reinforce change talk, and how to help others commit to a change plan appropriate to their stage of change. During the second day of training, the case managers will become familiar with the 4-session protocol and successfully complete a "mock" interview. We recognize that training is an ongoing process and follow-up booster sessions will continue after this initial training. For example, Dr. Osilla will take notes during each practice session and will provide supervision to the case managers on their adherence to the protocol and their use of MI techniques throughout the intervention delivery during the pilot. She will listen to every audio recording and provide ongoing weekly supervision and feedback/coaching to intervention case managers via phone to promote MI behaviors, to sustain MI proficiency, and to enhance intervention fidelity. Dr. Osilla has used similar procedures when training other counselors in teen court, DUI, and employee assistance program settings.

5A4 Interim Outcome Monitoring

5A4.1 *** Will interim outcomes be monitored in any way? For example, some intervention studies may have or be required to have a Data and Safety Monitoring Plan (DSMP) to assess that the intervention is not harmful and/or is having a positive effect.**

(●) **Yes** ( ) No

5A4.2 *** If "Yes", explain what will be monitored, when, how, and why.**
Dr. Osilla will monitor intervention delivery by providing supervision to the case managers on their adherence to the protocol and their use of MI techniques throughout the intervention delivery during the pilot. She will listen to every audio recording and provide ongoing weekly supervision and feedback/coaching to intervention case managers via phone to promote MI behaviors, to sustain MI proficiency, and to enhance intervention fidelity.

We will implement an Adverse Events protocol in the case that an adverse event occurs during the intervention delivery.

5A4.3 *** Does your funder or the RAND HSPC require a Data and Safety Monitoring Plan (DSMP) for your study? The RAND HSPC will make its determination during the review process.**
A.

5A4.4 *** Do you have the DSMP for this study ready to upload in draft or final form?**

(●) **Yes** ( ) No

5A4.5 You will be asked to upload the DSMP in section 7. A DSMP is typically done at the study level and you will be able to upload a single DSMP in section 7 and associate it with as many components as necessary. This is done to ensure that all components that should be covered are addressed in the DSMP. A DSMP is not the same as a DSP.

5A4.6 *** If "No", explain why this is not appropriate or necessary. For example, if the follow-up period is too brief to monitor interim outcomes or the intervention is benign or is determined to have minimal risk in the population being studied.**

SPECIFIC AIMS

Substance abuse and HIV infection are interrelated public health problems facing the homeless. An estimated 30-50% of homeless adults are alcohol or drug dependent[1,2], and homeless persons have been found to have rates of HIV infection 3 to 9 times greater than those with stable housing.[3,4] Housing First (HF) programs attempt to address the health problems facing homeless persons by providing housing to those that have been chronically homeless without required abstinence from alcohol and other drug (AOD) use. Hundreds of HF programs have been established across the United States since 1990.[5-8] These programs have demonstrated some success in reducing AOD use and sexual risk behavior in the short-term;[7,9] however, the evidence demonstrating their effectiveness is limited.[5,8] Approaches that are effective and easy to disseminate / implement are needed to enhance and prolong the positive behavioral effects of HF programs.

A number of recent studies from our research team and others suggest that the social networks of homeless individuals appear to influence their AOD use, recovery from co-occurring AOD and mental health disorders, engagement in HIV sexual risk behaviors, and even successful transition from homelessness.[2,10-15] As such, targeting the social networks of HF residents may be a promising approach to enhance the positive behavioral outcomes associated with these programs. The social environments of homeless individuals are multi-dimensional in composition and structure: they include a mixture of risky and non-risky influences and sources of potential support[11] and exhibit a range of structural features, from extremely dense to sparsely connected networks.[16,17] As such, social network-based interventions should assist homeless individuals in making compositional changes to their network (adding and/or removing types of network members) as well as structural changes (strengthening, weakening, adding connections among network members) in order to have a positive impact on their AOD use and HIV sexual risk behaviors.

We propose a 3-year study to develop and pilot test a social network intervention for permanent supportive housing (PSH) program residents in the Skid Row area of Los Angeles. The intervention will be developed for residents at Skid Row Housing Trust (SRHT), one of the largest HF providers in Los Angeles that has an ongoing successful collaboration with members of our research team. SRHT case managers regularly conduct support meetings with residents during the first 6 months of residency. The proposed intervention will enhance these supportive interactions. Case managers will be trained to deliver the 30-minute intervention sessions using a motivational interviewing style.[18] The motivational network intervention (MNI) will be conducted with the assistance of an electronic tablet and will involve five steps: (1) identifying members of a resident's personal social network and collecting information about network members (network composition); (2) asking the resident to rate the relationship strength of each unique dyad in their network in order to measure structural characteristics of the network; (3) displaying a network map that includes both structural and composition components of the resident's network; (4) discussing their social network's structure and composition in a non-judgmental and non-confrontational manner; and (5) exploring how the high-risk behavior in their social network impacts their own high-risk behavior and housing status. The MNI will be delivered across four sessions (baseline and two-week intervals). The proposed R34 application is in response to PA-10-013, which supports the preliminary development and testing of novel interventions. We propose the following aims:

Aim 1: To develop the MNI, train case managers to deliver the intervention, and test its feasibility and acceptability with 8-12 residents who have past-year harmful AOD use. We will iteratively beta-test an electronic MNI prototype based on feedback and refine/problem-solve recruitment/screening procedures prior to Aim 2.

Aim 2: To conduct a MNI pilot test with 60 residents with past-year harmful AOD use who are randomly assigned to receive the intervention or usual care. We will analyze baseline and post-intervention assessment data to determine the intervention's feasibility, acceptability and promise for a subsequent Stage 2 efficacy trial.[19] We will explore the following hypotheses: intervention participants, compared to controls, will show: a) increased AOD and safe sex-related self-efficacy b) increased readiness to change AOD use and high-risk sexual behavior, c) social networks with fewer and less central high-risk members and d) significant reductions in AOD use and fewer HIV sexual risk behaviors as compared to controls. *We will also examine trends in the data to explore associations between other proximal outcomes (greater efficacy, greater readiness to change, reduced riskiness of networks) and the distal outcomes (changes in high-risk behavior).*

At the end of this study, we will have an electronic tool for guiding case managers through a motivational social network interview with residents transitioning into a HF program and a well-described set of procedures for conducting the MNI. *We will also have preliminary data to explore hypotheses and estimate the variability and effect sizes of proximal and distal outcome measures for a future R01 efficacy trial with a mediation analysis.*

RESEARCH STRATEGY

Significance

This study is significant because: (1) Homeless individuals are among the most vulnerable populations in the United States in need of health interventions, in particular interventions that address their alcohol and other drug use (AOD) and HIV risk; (2) Social network interventions are needed to improve HF programs, which provide permanent supportive housing (PSH) to homeless individuals without requiring alcohol and drug abstinence; (3) Existing social network interventions are few in number, complicated to implement, and do not address the structural complexity of homeless personal networks; (4) Providing HF case managers with technology to easily present social network information to their residents may enhance care; (5) Exploring network visualizations in a motivational interviewing style may enable HF residents to develop strategies for intervening in their own social networks; (6) Developing computerized interventions increases the potential that evidence based treatments reach broader populations and that treatments are delivered with fidelity.

Homelessness is a significant public health problem leading to health consequences for vulnerable populations. Homeless individuals are among the most vulnerable to health problems in the United States. Homelessness is associated with a range of health problems, including chronic and infectious diseases[3,20-22] and mental health problems.[21] Among the most pressing health problems for the homeless are their high rates of AOD use, AOD dependence, and HIV risk. An estimated 30-50% of homeless are alcohol or drug dependent.[1,2] While AOD use is a leading cause of homelessness, AOD use is exacerbated by the stress of life on the street as well as exposure to high rates of AOD use among other homeless people. Exposure to HIV is also a significant problem for homeless people in part because of risky sexual behaviors associated with AOD use. Homeless persons have rates of HIV infection 3 to 9 times greater than those with stable housing.[3,4,23]

HF programs have had success at providing vulnerable populations with housing options. Because of the well documented association between health and homelessness, housing is often seen as an important health intervention.[24] There are two main approaches to providing housing to individuals who are chronically homeless: programs that make housing contingent on abstaining from AOD use and participation in treatment programs (“treatment first” (TF) approaches[5,25,26]) and HF programs, which provide housing without the requirement of abstinence.[7,8,27] Recent studies have demonstrated that HF residents have similar[28] or improved[7,9] AOD outcomes after 1-2 years compared to TF residents and had a 53% reduction in health service expenses compared to those on waiting lists.[29] The findings from the small number of studies directly comparing HF outcomes with TF approaches are promising and suggest that the requirement of abstinence prior to housing may be an unnecessary obstacle. Because of this promise, HF programs have become popular: since the first HF program started in the early 1990s, there are now hundreds of such programs.[6-8,30]

Addressing the role of social networks is a key to the success of HF programs. Although there is promise that HF programs can successfully provide housing without requiring abstinence from AOD, there are some concerns in the literature that HF is not necessarily a panacea to homelessness[8] and that it may be more helpful for those with less severe AOD addictions.[5] One concern raised is that those attempting to abstain from AOD use are inadvertently exposed to high-risk behavior because of the policy of not requiring abstinence.[8] HF residents may require support to face the challenge of entering a new social environment that includes a mixture of AOD users and non-users. A large and growing body of research demonstrates the importance of social networks to homeless and formerly homeless people and the association between social networks and the health behaviors and health outcomes associated with homelessness.[4,11,13,14] Social networks -- naturally occurring groups of people -- influence health and behaviors through social comparison, social sanctions and rewards, flows of information, support and resources, stress reduction, and socialization.[31-34] Social networks play positive and negative roles in the lives of homeless people.[35-37] Social networks provide social support, influence AOD use and HIV risk behavior, influence entrance into AOD recovery programs, influence recovery from co-occurring AOD and mental health disorders, and even influence successful transition out of homelessness.[2,7,10-15,38-40] The current study builds on these previous studies by providing formerly homeless HF residents and their case managers with precise information about the problems and opportunities in the residents’ social networks so that they can act as better informed agents of change. We will focus on developing a social network intervention for residents who are transitioning out of homelessness and into HF residency because their networks are likely to be undergoing a period of heightened volatility. Their recently improved housing status will put residents into frequent contact with new social influences and may also provide them with opportunities to reconnect with lost connections.

Most social network based interventions are either challenging to implement, expensive, or do not address the complexity in the structure of individual personal networks. There are limitations to

incorporating existing social network interventions into HF programs. Most existing network based AOD interventions are based on abstinence and/or focus on participants developing new social networks with other abstaining network contacts.[41-47] These interventions are only applicable to those who are already abstaining from AOD use. Network-based interventions also focus primarily on changes to the *composition* of social networks (the quantity and type of individuals in a network) and do not address the *structure* of social networks (the connections among the members of a social network).[48] Those that do incorporate both structure and composition focus on a narrowly defined group of individuals who share a risk behavior, such as injection drug use or men engaging in risky sex with other men, and the interventions target high-risk behavior among all group members.[49] Extensive ethnographic investigations are often required to identify group members prior to intervention implementation.[50-52] However, despite the prevalence of high-risk behavior among the homeless, the social environments of homeless adults are diverse and have a mixture of risky and non-risky influences and sources of potential support.[11] The social networks of homeless individuals also have a range of structural characteristics[16] and networks with more or less interconnections among high-risk and/or supportive network contacts.[17] There are no social network interventions that target the diversity of social contacts and social structure experienced by homeless people.

Social network interventions that use a personal network approach may be cost-effective and sustainable alternatives for HF and other programs. The personal network approach defines the composition and structure of a social network of one individual.[53] Personal network approaches have been used to study AOD use and HIV risk among homeless populations, including homeless women[10,11,17,54,55], homeless youth[56-63], and homeless men.[2,64,65] Personal network data differ from more traditional social network data (“complete” or “sociocentric”) because personal network data are collected from individuals about their perceptions of their own social environments rather than their connections to members of a particular group. Personal networks can be analyzed with data analysis techniques used for whole or complete network data.[53,66] The advantage of a personal network approach is that most interventions focus on delivering treatment to individuals rather than groups; therefore, enhancing existing interventions with a focus on personal networks is a way to include a focus on social networks without requiring a design that targets a bounded group. Understanding personal networks is important because the social network of one individual operates as a complex system with emergent properties that affect the focal individual.[67] One emergent property is the “social capital” available to take advantage of opportunities or call on in times of crisis.[13,68-70] Small changes in an individual’s personal network can have significant ramifications throughout the network. For example, a study of social capital and the social networks of formerly homeless individuals recovering from AOD use disorders showed how their networks shrank as they eliminated network ties associated with AOD use.[13] This made them more vulnerable to their networks fracturing after the death of a network tie.

The use of visualizations of personal networks may help HF residents become more aware of their existing social network system and provide insight into possibilities for change. A feature of personal network interviews is the ability to visualize the social network for one individual.[16,71,72] There are many ways to visualize personal network data with different strengths and weaknesses.[71] Personal network visualizations allow for display of both compositional and structural features. These visualizations are easy to understand and have been used with homeless participants with less than high school education.[16,62] Network visualizations are not new to health-related interventions. Visualization tools have been used by social workers to provide feedback to families about health and social support.[73,74] However, these techniques only provide information about network composition and do not incorporate structure. Network visualizations that incorporate structure have been used in a study of an educational intervention that promoted interactions among adolescents.[75] This study found that presentation of network visualizations prompted students to become strategic about how they formed connections to other students to improve their learning performance.

Using a motivational interviewing approach to presenting personal network information may help HF residents transition away from high-risk social networks and toward low-risk, supportive social networks. An intervention that incorporates Motivational Interviewing (MI) may be a logical vehicle for addressing maladaptive social networks. MI is an evidence-based intervention style[18] that has been used to reduce AOD abuse and HIV risk behavior.[48] MI moves people towards change through strategies such as expressing empathy (e.g., acceptance facilitates change), developing discrepancy (e.g., letting the patient present arguments for change), rolling with resistance (e.g., avoiding arguing), and supporting self-efficacy (i.e., a person’s belief in the possibility of change). Interventions using a MI approach can incorporate assessment and individualized feedback about a patient’s AOD use and related problems (e.g., motivational enhancement therapy).[76-78] Patients at early stages of change may receive information and feedback about their behavior; patients in later stages of change or who are already moving toward change may begin to

develop problem-solving strategies. Recently, one existing MI intervention has been enhanced with a social network component.[48] However, currently, there are no MI interventions that take into account both compositional and structural characteristics of social networks and no MI interventions targeting HF residents.

Developing an electronic tool for delivering a social network intervention to HF residents may provide a cost-effective way of disseminating evidence-based interventions to other HF programs. Electronic based intervention delivery tools offer low cost options for adoption of interventions in community settings and improve fidelity to intervention delivery procedures.[79] Use of an electronic tool to collect and present personal network information can also allow individuals to see their visualizations change over time, which may provide tangible evidence that the steps they are taking to make changes are having an effect, reinforcing the intervention's benefits. Computer-based interventions have been developed for HIV prevention[79,80] and AOD abuse.[81,82] Electronic tools developed to aid case managers and resident interactions could also be modified in the future to be self-administered, allowing for a greater number of individuals to benefit from the intervention.

Innovation

Our project is an innovative approach to enhancing the confidence and motivation of individuals to change their own social environments. Typical approaches to social network based interventions have used social network methods in the design, implementation and evaluation of health programs.[83] Our project is the first to give social network information directly to intervention participants so that they can act as informed change agents in their social environments. Most existing network interventions target members of a pre-defined group. Our intervention will allow individuals to target changes in their own personal networks that span across different types of network contacts and social worlds. The incorporation of visualizations of networks as mechanisms for providing easy to understand summaries of participants' social environments is an innovative approach to designing a social network intervention that targets change in both structure and composition of networks. Delivering information about social networks to intervention participants via MI style of interaction aided by an electronic tool that allows for consistent intervention delivery while remaining personally relevant to each participant is also an innovative aspect of our project. This is the first social network intervention to target HF residents; however, our innovative approach to designing a social network health intervention will provide a template for the development of other health interventions targeting diverse outcomes and populations.

Approach

Overview

We propose a three-year Stage 1 study[19] to develop and evaluate a MNI to enhance HF case manager support. The first year of the study will beta-test the electronic instrument for usability and acceptability of residents and case managers (Stage 1a). In the second and third years we will explore if the intervention is associated with reductions in AOD use and HIV risk behavior as well as social network changes compared to usual HF resident support through a small randomized controlled trial (Stage 1b). We begin by discussing the previous research that laid the foundation for this project and our conceptual framework and study design. We then describe our approach to addressing each aim.

Previous Studies

This project will be led by researchers in RAND Health, a division within the RAND Corporation. The team for the proposed study is interdisciplinary and combines strengths from medical anthropology, clinical psychology, social psychology, personality psychology, and statistics. The principal investigator is David Kennedy, a Social/Behavioral Scientist. Dr. Kennedy is a New Investigator and has assembled a team of experienced researchers in diverse and complementary areas for each study aim.

Exploratory social network research with homeless populations in Los Angeles. The foundation for this project comes from a series of exploratory studies conducted by RAND with homeless populations in Los Angeles County. Drs. Kennedy, Tucker, and Golinelli have collaborated for the past seven years on three different NIH funded studies of social networks and high-risk behavior of homeless women (funded by NIAAA), men (funded by NICHD), and youth (funded by NIDA). These projects involved multiple phases of data collection, including exploratory interviews about personal social networks. In the exploratory phases, respondents were asked to identify their primary social network contacts (referred to as network "alters") and report on the alters' characteristics as well as aspects of their relationships with each of them. For example, women identified family members, reported where they met non-family members, and reported if any of these alters were sex partners, drinking partners or drug use partners. They also rated each alter on the quality of their relationships with them and the likelihood that they would drink excessively, use illicit drugs, or engage in risky sex. Analysis of these data demonstrated that homeless women have larger and much more diverse social networks than previously thought and that their personal networks contained both high-risk and low-risk alters.[11] High-risk alters tended to be more central in the lives of women than higher quality relationships

(longer term, emotionally close, supportive). The results suggested a need to develop interventions to assist homeless women to strengthen their low-risk/high-quality relationships and extend the diversity of their networks to increase their exposure to positive and supportive influences.

Using network visualizations to explore the mechanisms connecting social networks with high-risk behavior. To further explore the mechanisms connecting social networks and high-risk behavior, we conducted brief exploratory interviews with homeless women[11] and youth[62] about their social networks. Based on their answers to network composition and structure questions, we displayed diagrams of their social networks using specialized software for collection, analysis and visualization[84] of personal network data.[85] Interviewers asked women to label the groups of alters that formed separate groups, or "components", with no connections to other alters. Homeless youth were also asked to name components as well as to identify alters who were most influential to their decisions about high-risk behaviors, such as drinking, drug use, and sex. Interviewers recorded the answers that the respondents gave by taking notes.

The figures below show three mixed-method diagram examples that were produced as a result of the exploratory interviews with homeless women and youth. The figures reveal interesting correspondence between high-risk behavior, social network composition and structure. In the network displayed on the left, the respondent identified four different types of alters. Two of these types were low-risk: her "Family" group and her "Sobriety Group". These alters did not interact with her high-risk "Party Group" or any of the "isolates"

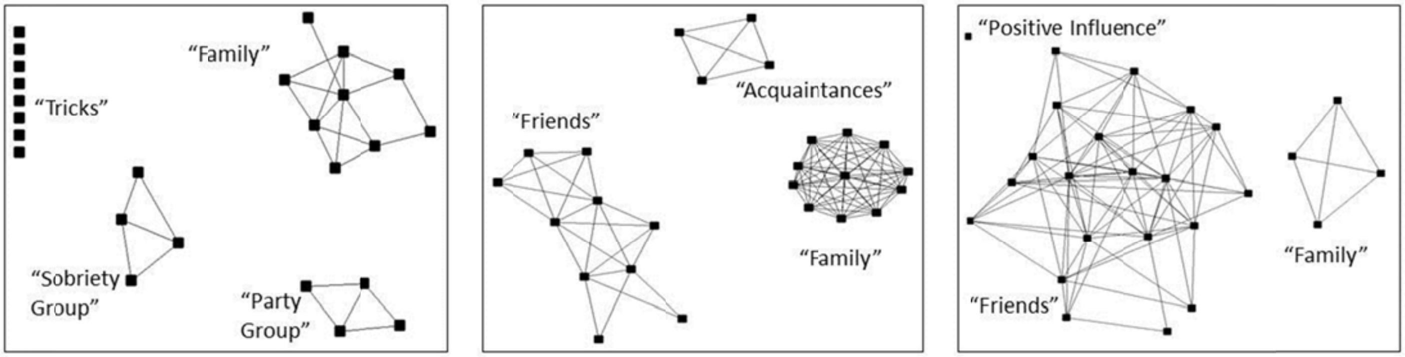

(those who have no connections to anyone else in the diagram) in the upper left hand side of the graph. Most isolates were described as "Tricks", indicating that the respondent occasionally had sex in exchange for money with these alters. The fact that no high-risk alters interacted with any low-risk alters is important because the respondent is free to engage in high-risk behavior with certain alters without her low-risk alters knowing. The respondent indicated that she used crack and drank alcohol heavily and the structure of her social network facilitates the continuation of this behavior despite her apparent interest in sobriety. The middle diagram represents a contrast to the diagram on the left. The respondent, who did not use drugs, had only one steady sex partner, only occasionally drank alcohol, and did not have any high-risk groups of network alters or any isolated network members. The respondent had three different alter groups who did not interact with each other. This network structure could potentially provide the respondent with a diversity of non-overlapping support. The diagram on the right depicts the social network of a male homeless youth who labeled one group of alters as his "Family" group and another as other homeless "Friends". He indicated that the one isolated network member discouraged AOD use. However, the respondent indicated that the alters in the "Friend" group frequently drank and used drugs and were likely to engage in risky sex. The isolated alter and the "Family" alters were very low-risk in comparison to "Friend" group. The small number of low-risk alters and their disconnection from the main group of network ties indicates that the influence these alters have on the respondent's behavior might be limited due to their lack of centrality to his overall network.

These exploratory studies influenced subsequent large-sample social network studies of homeless women, youth and men.[2,10,11,65,86,87] The conclusion from most of these studies is that social networks should be an important component of interventions targeting AOD use and HIV risk behavior among those dealing with homelessness. In a recent study of the perceptions of network visualizations, Drs. Kennedy and Tucker explored the idea that network visualizations could be used in social network interventions.[16] The goal of this study was to understand how those who are not experts in social network analysis perceive network diagrams. The results demonstrate that non-experts consistently identified structural features of social network diagrams. Our conclusion was that non-experts of social networks intuitively understand network diagrams, suggesting that lack of comprehension of network diagrams is not a barrier to using network diagrams in interventions.

Development of electronic tools to aid social network interventions. For the past 7 years, Dr. Kennedy has developed software to aid the collection and analysis of personal network data both as a

programmer of existing survey/personal network software and as a developer of new software tools. This builds on his previous experience as a systems analyst and programmer of electronic survey data collection instruments at a market research organization and at the University of Florida. Dr. Kennedy programmed the electronic personal network survey instruments for the exploratory studies of homeless women and youth described above and helped develop improved personal network collection/analysis /visualization software called Egoweb (available at http://egoweb.github.com) for the study of homeless men described above. The software is freely available and open source, meaning that it can be customized for any project with specific personal network data collection, analysis, and visualization needs. It has capabilities for displaying customized visualizations of personal networks and for collecting longitudinal personal networks. The software was developed in the java programming language and surveys can be administered through a web server. In the proposed study, Egoweb will be customized by the web development company bluSKY (see letter of support) for the specific needs of this project. The expertise of Dr. Kennedy is complemented by Dr. Osilla, who has 9 years of experience developing interventions that utilize MI, including computer-based interventions. Most recently, Dr. Osilla has led the intervention development and efficacy evaluation of a computerized brief intervention that incorporated motivational interviewing of first-time DUI offenders.[88] She was the main technical liaison between the intervention development team and bluSKY. Dr. Osilla is an expert in motivational interviewing and is affiliated with the Motivational Interviewing Network of Trainers.

Randomized controlled trial development and evaluation. Drs. Osilla and Hunter also have collaborated on NIAAA- and NIDA-funded AOD use treatment grants and a NIDA-funded project to develop and test a group motivational interviewing AOD intervention.[26,89-91] They have evaluated them using quasi-experimental and randomized clinical trial approaches. Dr. Hunter currently collaborates with Skid Row Housing Trust (SRHT) on two SAMHSA-funded projects that provide AOD abuse treatment and other supportive services to residents in eight SRHT buildings (see Hunter biosketch and SRHT letter of support). On these projects, chronically homeless individuals that screen positive for a mental health and/or AOD disorder and are transitioning to housing are given access to onsite mental health and substance abuse treatment services. Dr. Golinelli will lead the randomization strategy for this project and the pilot data analyses. She has extensive experience developing sampling designs and randomization/recruitment strategies for homeless populations and has been the statistician on large randomized controlled trials and will lead the selection of appropriate analyses procedures for the proposed study.

Conceptual Framework

The proposed MNI is grounded in social network theories (complex systems and social capital theories) and theories central to the MI approach[18,92] (self-determination, psychological reactance, and self-efficacy theories) (see Figure 1). The MNI will target social network structure and composition and the relationship of these to high-risk behaviors, such as AOD use and HIV risk behavior. Complex Systems Theory as applied to social networks assumes that a set of social relationships between individuals in a group has emergent properties that would not be apparent in an examination of the individual parts of a larger social system.[66,67,93] The theory also suggests that changes made in one area of the system may have effects that flow throughout the rest of the system and that approaches to change should consider the potential impact on the whole system. Social capital theory identifies an emergent property of the social resources in a network, such as information channels, norms and expectations/obligations that impact people's lives.[69,93] Social capital can have positive or negative impacts on health and network resources are either amplified or dampened depending on how and with whom links are formed.[13,68,70] The theory of self-determination emphasizes patient autonomy and innate capacity for growth and change.[94,95] Self-efficacy theory indicates that people with more confidence in their ability to change their behavior are more likely to change.[96] Psychological reactance theory posits that when people feel undue pressure to give up a particular action, they feel that their freedom is threatened. Together, these

Figure 1. Conceptual Framework

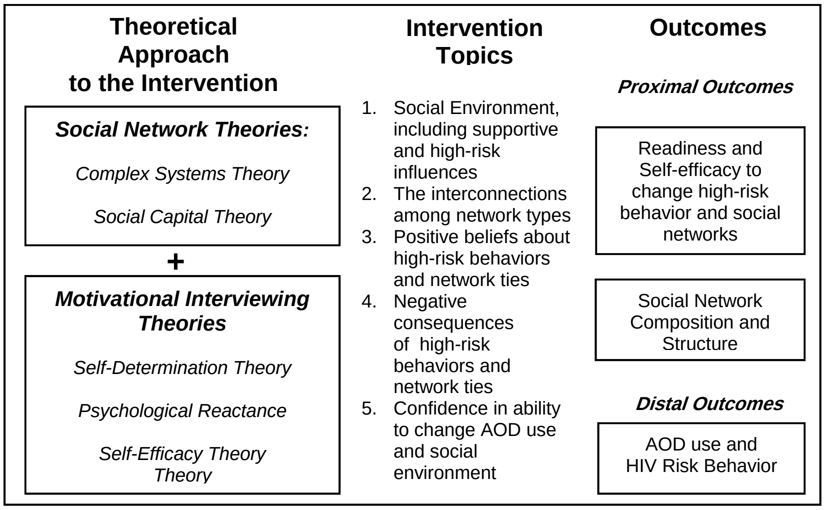

theories suggest that an intervention that presents HF residents with personalized network information using a MI style may empower them to change their social environment. For example, we will train case managers to introduce the MNI by emphasizing autonomy with statements such as “The goal of this interview is to give you information so that you can better understand and make decisions about your social life. What you do with this information is totally up to you.” To build self-efficacy, interviewers will be trained to identify statements of change and to encourage change talk by asking residents to explain their reasons for wanting change and to reinforce this through encouragement. Case managers will also ask residents to talk about practical steps they can take to initiate changes in their social networks. This strategy is consistent with MI by eliciting and strengthening confidence talk or the language associated with self-confidence to change. Having the resident verbalize his/her reasons for change and the steps to initiate change may reduce perceived barriers to change and increase self-efficacy. We plan to use beta-testing in Aim 1 to adapt these strategies for this population (e.g., encouraging change, presenting different network diagrams that better demonstrate problem areas in the network, generating feedback from case managers about what has worked for them in the past).

Description of Intervention

The MNI will consist of four sessions that build on each other. Previous in-person MI studies with other populations have typically been 1-2 sessions.[97-101] The four MNI sessions will allow enough time for network change strategies to be put into place and for residents to see changes that can be encouraged by case managers. The first MNI session will occur within two weeks after SRHT case managers complete the initial needs assessment (within 30 days of resident move-in) and after the baseline survey. The MNI sessions will last approximately 30 minutes and will consist of two parts: a network interview with closed-ended network questions that cover the time period since their last interview (see Appendix A) and a discussion of a resulting network visualization conducted in a MI style (see Appendix B). The network interviews are based on interviews we conducted with homeless men and women, many of whom had low education levels (25-33% without high school education) and do not require respondents to be literate. In the first session, case managers will read network questions from the MNI electronic tablets verbatim. These structured network interview questions are an abbreviated set of the questions asked at baseline (described below), will cover the timeframe between the baseline and first session (or between visits in subsequent sessions), and will consist of a network name generator (e.g., “List 10 people you have interacted with in the past 2 weeks.”), network composition (e.g., “Is __a man or woman?” “Have you drank alcohol with ___?”), and network structure questions (e.g., “Does (Alter 1) know (Alter 2)?”). Answers to these questions provide raw data to generate network visualizations. To identify the key recent social experiences in their lives, participants will be asked to name 10 network ties who they have interacted with in the past two weeks and who have been significant in their lives, either positively or negatively. Participants will define significance for themselves but case managers will suggest that they consider those who have had an important emotional, social, financial, or other influential impact. Once case managers have elicited this list of 10 alters, they will confirm if any alters were named in the baseline assessment network interviews. The MNI will be synchronized with a network server and will be pre-loaded with each participant’s baseline network data and case managers will link the names that have already been named by the participant. Case managers will ask some basic questions about each of the alters who were not previously named, (e.g. gender, if the alter is a relative or not). The advantage to the electronic interface is that questions can be skipped for any alters who were mentioned in previous interviews to avoid re-asking questions about characteristics that do not change between interviews. For all alters named, case managers will ask participants a series of questions rating their recent relationship with the alter. For example, questions include how emotionally close the participant felt to the alter, how often they interacted, if they had sex with the alter and if they used a condom, AOD use with the alter, their supportive or negative interactions with the alters and if they want to spend more or less time with the alter in the next 2 weeks. Based on our previous network interviews, we estimate that this process will take about 15 minutes to complete

Once all network questions have been asked and answered, case managers will lead a discussion about the participant’s network in a MI style guided by the network visualizations (Appendix B). They will show the participant a series of 3-4 network diagrams customized for the participant. The initial diagram will present the network structure and subsequent diagrams will highlight positive relationships (e.g. emotionally close, supportive), negative relationships (e.g., argumentative relationships, negative emotions associated with the alter) and relationships that involve high-risk behavior, such as AOD use or HIV risk behavior. Case managers will explore the pros and cons of residents’ current social network composition and structure and discuss their readiness, willingness and confidence to change things about their networks that they do not like, such as spending more or less time with certain network members or introducing network members to each other. Discussions will especially focus on relationships with sex partners and alters who either discourage or foster

engagement in high-risk behaviors that lead to undesirable outcomes. *Case managers will ask participants about their behaviors with these alters (safe sex decisions, AOD use), how their relationships with these alters impacted decisions about doing things they know put them at risk either currently or potentially in the future, and what they may want to change about their behaviors and relationships.* They will also look for opportunities to encourage discussion of strategies/tactics for change that may take place or begin to take place over the upcoming two week interval and to set goals for themselves. For example, if the participant has not had any positive relationships, a case manager can ask the resident, "Is there someone you haven't named who you would like to interact with more? What are some steps you can take to interact with ____ in the next two weeks?"). Case managers will print out network diagrams during the interviews and give these to the resident. In addition to depicting the network, the printouts will have case manager notes about the statements the resident made regarding their goals and the steps they are willing to take towards modifying their social network relationships over the next two weeks and will have space for residents to write their own notes.

The three subsequent sessions will also cover the time periods between sessions and will repeat the network interview and discussion. Discussions of the visualizations will focus on the ways in which the network changed between sessions, including discussing alters they either started or stopped interacting with since the previous MNI session and discussing what has not changed since the last interview. Case managers will review notes from previous interviews and will ask follow-up questions about attempts at change and encourage steps that residents are taking toward meeting their goals. The diagrams printed during each session will enable participants to continue contemplating their goals for change. The in-depth discussions of the social environment of the resident and the documentation of the network composition/structure and stated goals about change will enhance the supportive relationship *between the case manager and resident* beyond the individual MNI sessions. The network visualizations/printouts resulting from the network interviews will provide both case managers and residents with a tangible output on which to focus discussions and make abstract discussions of social life, relationships, and social change more concrete. *They will also provide documentation of the personal goals and aspirations of the residents across sessions and facilitate discussions of successful attempts at change to allow for reinforcement during MNI sessions and other interactions.*

Study Design

Overview We propose a 3-year study to develop and pilot-test a MNI for high-risk behavior among residents of a HF program with *past-year harmful AOD use (including dependence)*. For Aim 1, we will recruit residents of SRHT who have completed their first six months of residency. We will conduct iterative MNI program beta-testing with residents (n=8-12) and case managers (N=4). In Aim 2, we will pilot-test the MNI with SRHT residents who have been residents for less than 30 days and have completed the intake interviews that are given prior to undergoing 6 months of weekly case manager support. We hypothesize that residents receiving the MNI (n=30) will have increased AOD and safe-sex-related self-efficacy and readiness to change and networks with fewer and less central high-risk network members compared to residents receiving usual PSH case manager meetings (n=30). We will also monitor other distal outcomes that we expect would eventually result from the MNI but may require more time than we can observe in the pilot test period. For example, we will monitor if MNI participants have decreased AOD use or have fewer HIV risk behaviors.

Setting. We build on our ongoing collaborations with SRHT (see Letter of Support and attached Housing Retention and Stability Report -- Appendix C). SRHT is one of the largest providers of PSH in the United States, and, in particular, the Skid Row area of Los Angeles, which has the largest concentration of homeless adults in Los Angeles County. SRHT operates 22 PSH buildings with over 1,700 individual units,14 of which provide housing plus support to residents.

Study Participants *Study participants will be 72 (8-12 for Aim 1 and 60 for Aim 2) adults aged 18 or older who are (1) receiving PSH (2) speak and understand English, (3) are not cognitively impaired (either by identifying those having a diagnosis of dementia in the SRHT intake interview or using the Short Blessed Scale Exam[102]), and (4) screen positive for past -year harmful AOD use using the AUDIT-C (a score > 4 for men and > 3 for women)[103] and DAST (a score greater than 2).[104-106]*

Recruitment As part of entry into HF, case managers conduct an intake assessment with new residents. The intake is initiated within the first 30 days of entering housing. For this study, case managers will be trained to ask any new resident that speaks and understands English and meets the study eligibility criteria (i.e., no clinical diagnosis of dementia) if they are willing to be contacted to be part of this study and how best to be contacted. The resident's consent will be limited to agreeing to be contacted about the study and giving his/her name and contact information to the research team. If the resident agrees to be contacted, a research team member will contact the resident, describe the study, obtain consent for the study screener and administer the screener. If the resident screens eligible, the resident will be invited to participate in the study.

Signed informed consent will be obtained prior to the baseline survey.

Flow rates. Using data from 2010 (Appendix C and D), we estimate the number of individuals entering HF units to be 15 per month and 92% (14 residents) will screen as eligible for harmful AOD use based on intake data from current SRHT case management records. Given our goal to enroll a total of 60 residents for Aim 2, we have estimated a six-months recruitment window. Our previous community research experience has shown that flow rates may differ once study funding is in place. We are conservative in estimating the number of people who will agree to be a part of the study. The advantage of our Aim 1 design is to beta-test our recruitment procedures and flow rates. Thus, if we find that we are overly ambitious in our estimates, we can adjust our timeline and procedures accordingly by extending our recruitment period into Year 3 of the study.

***Measures*.** These measures (see Table 1) were chosen because they have been used in various populations, including low income, low education AOD using populations[107,108] and populations engaging in heavy AOD use and high-risk sexual behavior.[109] The measures have also been recommended by the VA Mental Health QUERI taskforce[110] and NIDA.[111] *The limited scope and resources of this feasibility study limits the number of follow-up assessments to one. We will assess participants within 2 weeks after the final MNI session in order to measure the immediate benefits of the intervention rather than longer-term effects*. Measures will be collected in a one-on-one interview with a RAND research assistant using computer-assisted personal interviewing (CAPI) software*. Interviews will last between 45 minutes and 1 hour.*

Table 1. Proposed Participant Measures and Administration Timeline

	Screening	Baseline	*Post-MNI*
At-Risk Drinking: AUDIT-C	X		
Any Illegal Drug Abuse: DAST	X		
Demographics: Age, Gender, Race/Ethnicity, Education, Treatment History		X	*X*
Readiness to Change: RTCQ, RTCQ-SB, Ladders		X	*X*
Self-Efficacy: AASE, Condom Efficacy, Ladders		X	*X*
AOD Use: Timeline Followback, Oral Fluid AOD validity test		X	*X*
High-risk sex: Unprotected sex, concurrent partnerships		X	*X*
Social Network Measures: Composition and structure of drinking/drug/abstinent supportive network members		X	*X*
Client Satisfaction Questionnaire (MNI Participants only)			*X*

Baseline Measures. We will incorporate baseline **demographic characteristics** (age, gender, race/ethnicity, education, income, time homeless) collected at screening and collect additional information about AOD use and mental health treatment to explore as potential intervention effect moderators.

Outcome Measures*.* We measure both proximal and distal outcomes. Our proximal outcomes are readiness and self-efficacy to change AOD use and safe-sex behavior, and social network composition and structure. Our distal outcomes are AOD use behaviors and HIV risk behaviors. For our proximal outcomes, we will measure readiness to change in two ways. The first method is a one-item **Contemplation Ladder** that displays a picture of a ladder and corresponding stages of change on a 0-10 scale (e.g., “Starting to think about how to change my drinking/drug use/condom use patterns”). These measures have adequate reliability and good predictive/concurrent/discriminant validity[112] and have been used for smokers with schizophrenia[113], drinking adults with multiple sex partners[109], and welfare applicants.[107] We will also measure readiness to change using the **Readiness to Change Questionnaire (RTCQ)**,[114-116] which consists of 12 items that assess the readiness to change AOD use (Precontemplation, Contemplation, Preparation, or Action) and the **Readiness to Change Risky Sexual Behavior (RTCQ–SB)** scale**,** which consists of 11 items adapted from the RTCQ for safe-sex behavior.[109] We will measure self-efficacy using two items that assess the participant’s **importance and self-efficacy** to quit smoking[113] on a 0-10 Ladder scale, and adapt these two items for AOD use, safe-sex, and network changes. We will also use an abbreviated version of the **Alcohol Abstinence Self-Efficacy Scale (AASE)**,[117] which consists of 4 items indicating how confident participants feel in their ability to abstain when depressed, relaxed, craving substances, and offered alcohol. We will also measure **Self-Efficacy to Use Condoms** which has shown good reliability and concurrent validity[118] and has predicted unprotected sex in homeless populations.[54,62,65]

We will collect **social network** data similar to our previous homeless studies to measure change in network composition and structure.[2,10,11,54,55,58,61] Participants will be asked to name a larger number of alters (20) during baseline and post-intervention assessments than during MNI sessions because a minimum of 20 alters is necessary to produce unbiased estimates of network structure[119,120] and to measure network

composition[11] (but not necessary for discussion with case managers). Participants will be asked demographic questions about each of these network alters (age, gender, if relative or not, their drinking/drug use/safe-sex behavior), questions about their relationships with these alters (emotional closeness, frequency of contact), and about the relationships between alters (e.g. "Does (Alter 1) know (Alter 2)?"). We will construct measures that are similar to measures of **Perceived Social Support**[15,121] (the number and/or proportion of network members who provide support) and measures that result from the **Important People Drug and Alcohol interview**, in which network members are rated on relationship characteristics (e.g. emotional closeness), drinking, drug use, safe-sex behavior, and their approval/disapproval of the respondents' high-risk behavior.[122]

For our distal outcomes, we will use self-reported drug and alcohol use as our primary AOD use measure assessed by the **Timeline Followback** (TLFB). The TLFB is a calendar-structured interview that provides memory cues to assist in accurate recall of AOD use and sexual behaviors.[123] It has excellent reliability[161] and validity.[162-165] At each assessment, past 90-day drinking (drinks per drinking day and frequency) and drug use (frequency) will be assessed. We will consult SRHT intake data, which include recent drug use and drug use history, to select the most commonly used drugs among residents. Frequency and intensity of use are thought to represent independent dimensions of AOD use behavior and tend to be relatively uncorrelated, making them a good choice as outcome measures.[166] We will derive a Quantity Frequency Index (QFI), which has a history of reliability and validity across various populations.[124] *We will use an Oral Fluid test to validate AOD use self-reports. These tests screen for recent alcohol, THC, opiates, amphetamines, cocaine, and benzodiazepines with good sensitivity and specificity while being less invasive and resource-dependent than urinalyses.*[125] To measure **HIV risk behaviors**, we will ask about the respondent's sexual behavior in the past 90-days overall and with particular partners they name as alters. We will ask them how many times they had sex (vaginal or anal) and how often they used condoms when they had sex. We will also ask if they were engaging in sex with other partners concurrently. We have used these measures in studies of HIV risk among homeless women[54], youth[62], and men.[65,126] To measure network changes, we will construct measures of how central various network members are in the network using standard social network centrality measures.[53,66] We will calculate these measures for alters who are either supportive of high-risk behavior or not and compare changes in these measures from baseline. We will also calculate measures of overall structural characteristics, such as density or centralization, to determine if the intervention affected overall interaction among network members.

We will also use the **Client Satisfaction Questionnaire** (CSQ-8), a standard, easily administered, and reliable 8-item Likert-type measure of client feedback regarding services provided in a treatment program.[174,175] This questionnaire has been used in studies with community mental health patients.[176,177] *During the post-intervention assessment,* MNI participants will complete a brief **satisfaction survey** adapted from previous studies.[127-131] Participants will answer 8 questions, including "I would recommend this interview to a friend" and "The information I received will cause me to change my relationships." Two open-ended questions will ask participants to state what was most and least useful about the interview.

MI Fidelity Measure. As in our other intervention work[88,132,133], we will measure fidelity to the intervention protocol with an 8-item scale to determine if key components of our intervention were discussed. The checklists consists of a 5-point Likert scale with response options ranging from "completely", "partially", to "not at all" covered. For example, items will assess whether the case manager discussed the client's desires to interact with network ties more/less frequently, etc. The audio recording of each session allows us to monitor the fidelity of each facilitator. A research assistant will complete these forms and Dr. Osilla will review them.

Aim 1: Develop and Beta-Test the network intervention

The primary purpose of Aim 1 is to finalize the MNI electronic tool using an iterative beta-testing process based on feedback from residents and case managers. This includes modifying the existing Egoweb software into the MNI prototype, beta testing the prototype with case managers and residents, analyzing data from beta test sessions, and making additional modifications to the MNI. ***Developing MNI prototype***. We will work collaboratively with bluSKY to iteratively develop specifications for the modifications of Egoweb into the MNI and evaluate these modifications. This includes drafting "storyboards" or intro and exit MNI screenshots and iteratively revising based on resident feedback. First, we will work collaboratively with bluSKY (see letter of support) to modify the Egoweb software that will be used to guide case managers through the MNI with study participants. The main Egoweb software functions – guiding interviewers through questions that elicit names of network alters, questions about each of these alters, questions about the relationship between unique alter pairs, and presentation of network diagrams – currently operate on electronic tablets and do not require modification. Based on feedback from beta-testing (see below) we will modify screens to improve usability for case managers and residents. Second, network visualization features will be enhanced to facilitate interaction

during the interview and changes over time. Modifications may include alternate views of networks that case managers can choose in the moment based on feedback with participants during interviews or enhancement of touch-screen capabilities to allow for the interviewer to touch an individual node on the graph (a network alter) and enter notes about actions specific to that network member that the resident plans to take over the two week period between MNI deliveries. Finally, the MNI will require a customized front end set of screens and set of exit screens and webserver hosting setup. At the end of development, we will program the MNI (see Appendix A and B for draft examples) and have a live prototype MNI to beta-test with residents (Milestone 2 – See **Study Timeline and Milestones** section).

Beta-testing prototype. We adapt usability testing procedures assessing ease of use and enjoyment from previous informatics work[88,128] for two phases of MNI beta-testing. The beta-testing purpose is to assess resident and case manager experiences when interacting with the MNI tool and to make changes to the tool based on this feedback to improve comprehensibility and ease of use.[134] We will revise the MNI on an ongoing basis in order to implement any suggested changes before we proceed with the next beta-test. In the first phase, RAND will focus on improving the residents' experience with the tool. We will conduct the MNI with residents using a MNI prototype and interview them about acceptability and feasibility. We will coordinate with SRHT to recruit 4-6 residents to review the MNI prototype. Drs. Kennedy, Osilla, and Hunter will conduct these initial sessions. The interviews will be audio-recorded with the resident's consent. After each intervention we will ask residents about their experiences (e.g., what your overall impressions of the session were; what was most helpful/unhelpful; what did you like/dislike/would like change). We will also walk through the MNI screens with residents for feedback and explore if the wording or screen visualizations are difficult to understand. In the second phase, we will beta-test the MNI with both residents and case managers. Dr. Osilla will train 4 case managers to deliver the MNI to 4-6 residents (see Training below). RAND will then independently interview residents and case managers about their experience. The purpose of this phase of beta-testing is to further assess the residents' experience and to assess case manager's experience (e.g. ease of table use, navigation through screens, what errors are common, how easy is it to correct these errors) when interacting with the MNI tool. We will also assess perceived barriers to using the MNI during case management sessions to identify potential barriers for Aim 2 (e.g., what problems might we face if case managers had to use the MNI with several different residents? What are some ideas for solving those problems?).

Residents will receive $25 for beta-testing and we will compensate SRHT for training and beta-testing time. While we expect the MNI to be easy to use and understand, we will use this phase to problem-solve ways to make the MNI easier to use and helpful. If we find that the MNI is still difficult to understand after beta-testing with 8-12 residents, we will revise again and beta-test with additional residents until the finalized MNI is ready for pilot-testing. We realize this may extend our study timeline and therefore devote Year 1 to develop the MNI. At the end of beta-testing, we will have a finalized MNI ready for our pilot test (Milestone 3).

Training. Training is consistent with our previous studies and will include a 2-day workshop that will cover two areas: Motivational Interviewing (MI) and the social networking interview protocol. The MI training will follow the eight stages in learning MI[135] including building motivation to change and strengthening commitment to change.[136] We will focus on foundational elements of MI and the spirit or counseling style that underlies its techniques. The training will involve a mix of didactic presentation, demonstration, and several practice sessions involving role playing exercises that are geared toward people with little or no exposure to MI. Through this training, they will learn: key principles of MI, an empathic, yet directive, counseling style, semi-structured techniques to help people talk about change, how to "roll with resistance" with reflections, how to use information and feedback as a motivational tool, how to reinforce change talk, and how to help others commit to a change plan appropriate to their stage of change. During the second day of training, the case managers will become familiar with the 4-session protocol and successfully complete a "mock" interview. We recognize that training is an ongoing process and follow-up booster sessions will continue after this initial training. For example, Dr. Osilla will take notes during each practice session and will provide supervision to the case managers on their adherence to the protocol and their use of MI techniques throughout the intervention delivery during the pilot. She will listen to every audio recording and provide ongoing weekly supervision and feedback/coaching to intervention case managers via phone to promote MI behaviors, to sustain MI proficiency, and to enhance intervention fidelity.[137] Dr. Osilla has used similar procedures when training other counselors in teen court[26,90], DUI[88], and employee assistance program settings.[130]

Analyses. Aim 1 data analysis will be conducted on notes taken during and immediately after interview sessions. Interviews will be audio recorded with Livescribe "smart-pens" (www.livescribe.com), which we have used to produce audio recordings and hand-written notes in exploratory interviews with homeless men and youth. Notes will be taken during interview sessions and will be enhanced with additional notes after interviews

are completed. The data collection and analysis procedures will follow approaches we have used in our previous exploratory qualitative analysis and intervention development work.[88,90,130,131,138-144] We will conduct open coding on notes to identify themes and project team members will discuss and come to consensus on a final set of themes. Quotes illustrating each theme will be tagged in Atlas.ti and we will write a final summary description of each theme. This analysis will be performed for the purposes of understanding both the MNI feasibility and acceptability and informing the delivery of MNI with SRHT case managers. The Aim 1 data analysis will also aid additional modifications to the wording, order, and presentation of MNI screens and questions as well as additional refinement of network questions and visualizations.

Aim 2: Conduct a Randomized Controlled Pilot Test of intervention

Overview. In Aim 2 we will pilot-test the MNI intervention with a sample of 60 SRHT residents. We will enroll the residents from 14 SHRT buildings that are designated as SPC (shelter plus care) buildings where clients must meet the Housing and Urban Development's criteria as chronically homeless. Thirty of the 60 enrolled clients will be randomized to the intervention arm and 30 to the control (care as usual). SRHT will recruit 8 case managers to deliver either the intervention or usual care. Four of the 8 case managers will be randomly selected to be trained to deliver the MNI. The other case managers will not have access to tablets running the MNI and will deliver usual care to the controls. All new residents who agree to participate in the study will be assigned to one of these 8 case managers depending on to which study arm they are assigned.

Data Collection. Potential participants will be recruited from initial intakes conducted by case managers at SRHT (Figure 2). The RAND research team will monitor the number of move-ins and intakes conducted to ensure that every adult resident is screened for eligibility and offered the opportunity to participate. Residents who meet study criteria and agree to become participants will be asked to complete a baseline assessment interview and a _post-intervention interview during the two weeks following the end of 4 MNI sessions_, Residents will be paid $30 for the baseline assessment and $40 for the post. Eligible residents will be randomly assigned to MNI or usual case manager support. _Those assigned to the MNI group will also be asked questions assessing client satisfaction during the post-intervention interview._ All residents, regardless of their participation in MNI or whether they are continuing to be residents at SRHT will be followed.

Figure 2. Intervention and Data Collection Flow

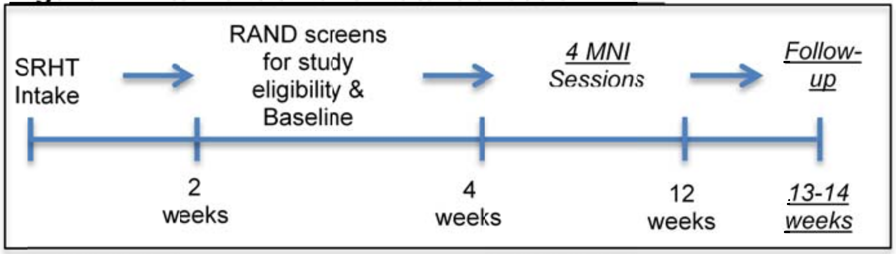

Randomization. Residents will be randomly assigned to usual case manager support or case manager support + MNI after their baseline interview using a stratified permuted block randomization strategy. RAND will be responsible for this step and will inform SRHT of the randomization results. Although we will randomize the participants to one of two groups, given the smaller sample size, the randomization might produce an unbalanced assignment with respect to important characteristics. _For this reason the randomization will be stratified with respect to gender and by AOD dependence status. About 26% of SRHT clients are women and about 32% are AOD dependent and stratifying these characteristics will ensure an approximately equal number of women and residents with AOD dependence assigned to both conditions._ If the intervention and control groups turn out to be unbalanced with respect to other characteristics, we will use covariate adjustment at the analysis phase. The randomization sequence will be generated by Dr. Golinelli and kept hidden from the other team members so that the assignment to the two conditions cannot be gamed.

Usual Care. After intake, residents meet with case managers regularly for up to 60-90 days (based on need). Meetings last 15-60 minutes and are customized to the residents' needs. Case managers facilitate residents' access to on-site services, such as support groups, assessments, treatment planning, etc.

Tracking Information. SRHT will assist with tracking of participants. Case managers keep information about the small number of residents who leave prior to one year of residence and will assist locating any non-residents for the post-interview. RAND has extensive experience with tracking and locating similar populations and will collect information at baseline to assist contacting participants for the post-interview.

Attrition. A concern for Aim 2 is attrition given the sample size. However, we have an excellent record in achieving good response rates with homeless populations and our work with SRHT suggests response rate will be high. We conservatively estimate a response rate of at least 85% for post-intervention interviews.

Aim 2 Data Analysis and Power

Overview. The major goal of Aim 2 is to assess: the intervention feasibility, acceptability and promise, estimate the variability of measures in the study population, and obtain preliminary estimates of intervention effect sizes. Given the small sample size, sophisticated modeling and adjustments for non-response might not be possible. Given the stated goal for this aim, the analyses will be primarily descriptive. We will estimate the outcomes' variability in this population and more generally assess the hypothesized trends to determine the intervention promise <u>*and identify potential mediators for future study*</u>.

***Intent-to-treat sample and intervention non-compliance**.* Analyses will use the standard intent-to-treat (ITT) approach to examine the effect of offering the MNI to all incoming residents. We will attempt to follow up with all residents, regardless of their MNI participation. Our ITT approach will analyze residents as belonging to the group they were randomized to, regardless of their compliance, because excluding those who do not complete the MNI would bias results in favor of MNI, increasing type I errors.[145]

Analysis plan. As stated above this is a pilot study and we may not have sufficient power to demonstrate statistically significant effects or fit sophisticated models. However, we will be able to obtain intervention effect estimates on both the proximal (efficacy and readiness to change AOD, safe-sex behavior, and networks) and distal outcomes (AOD use and HIV risk behavior) using a difference in differences (DID) approach.[146] The DID approach is well-suited to the data generated by the adopted study design: for every subject we will have a pre- and post-intervention observation and a randomized group indicator (intervention or control). We will implement the DID approach by fitting a mixed-effects model in which subjects are treated as random effects while time, the intervention group indicator, and their interaction are treated as fixed effects. This modeling approach accounts properly for the two repeated measures' correlation on each subject and tends to produce efficient intervention effect estimates.[147] The intervention effect estimate is given by the coefficient of the interaction between the time and intervention indicators. <u>*We will analyze oral fluid AOD use results with procedures we have used in previous studies: if oral fluid results are discrepant with AOD use self-reports, we will explore recoding using predictive mean matching by predicting the outcome at follow-up for all clients and for each client who screened positive imputing his/her number of problem substance use days by taking the value from the client with the closest predicted value.[148,14]*</u>

Power considerations. In order to obtain preliminary estimates of intervention effect sizes and to conduct a power analysis for a possible future study, we will take into account the error associated with the estimates of effect sizes, since such estimation error (which tends to be quite large) may lead investigators to enroll inappropriate sample sizes for future studies.[150150] More specifically, to determine the sample size for the larger study we will determine what the clinically meaningful effects are for somewhat similar interventions from the literature and we will standardize such effects using the variability estimates obtained with our pilot data.

Limitations. The main limitations of this study include the following: 1) A small sample size limits our ability to detect statistically meaningful differences in our outcomes; 2) Greater participant understanding of their networks may influence how they answer questions about their networks in the post MNI assessment, limiting our ability to separate changes in networks from changes in perceptions of networks; 3) The intervention focus on social networks may have unintended effects on the larger group of SRHT residents; and 4) The completion of the baseline network interview may also have unexpected effects on the control group. Insights from this feasibility study will inform how to overcome these limitations in a large sample Stage 2 effectiveness study.

Study Timeline and Milestones. We will target completion of the following milestones: 1) We will work closely with SRHT on implementation plans so we can ensure the feasibility of beginning recruitment and intervention implementation without setbacks; 2) During Year 1, we will develop and beta-test the MNI; 3) Early in Year 2, we will finalize the Aim 1 activities, including finalizing the MNI and 4) developing the assessment instruments; 5) In Year 2 we will complete enrollment of 60 residents and begin the finalized MNI pilot test and finalize Aim 2 data collection; 6) During Year 3 we will analyze assessment data, and 7) disseminate the results with a manuscript for publication and a conference presentation; and 8) If the MNI demonstrates promise for improving outcomes, we will also develop an R01 application for a Stage 2 effectiveness trial of the MNI.

Conclusion. The proposed study addresses a critical public health problem: how to address AOD use disorders in a chronically homeless population transitioning to a housing program. The MNI is innovative in that it develops technology to assist individuals to change their own social networks while improving case managers' ability to support these changes. This study will provide the foundation for a larger trial that will address the current study's limitations with a larger sample, a longer follow-up assessment, controls on unintended network effects, and measures of high-risk behavior and network changes that do not rely on self-reports. If successful, this project may provide a template for the development of social network based health interventions that can target a diverse array of outcomes and benefit a wide variety of populations.

PROTECTION OF HUMAN SUBJECTS

The study will undergo review by the RAND's Human Subjects Protection Committee (HSPC) if funded. The HSPC serves as RAND's Institutional Review Board (IRB) to review federally funded research involving human subjects, as required by numerous federal departments and agencies, including the Department of Health and Human Services (HHS). RAND's Multiple Project Assurance of Compliance (#M1031) with HHS regulations (45 CFR 46) also serves as our assurance of compliance with the regulations of other federal departments and agencies. All research projects, whether or not federally funded, are reviewed by RAND's HSPC in accordance with HHS regulations.

Risks to the subjects

Human subject involvement and characteristics:
Participants in the current study will be residents of Skid Row Housing Trust (SRHT). Study participants will be 72 (8-12 for Aim 1 and 60 for Aim 2) adults aged 18 or older who are (1) receiving permanent supportive housing (PSH) and are within 30 days of move-in at SRHT (Aim 2 only) (2) speak and understand English, (3) are not cognitively impaired (using the Short Blessed Scale Exam99), and (4) screen positive for past -year harmful substance and alcohol use. The study will exclude persons with a diagnosis of dementia, as noted in their SRHT intake interview.

This study is responsive to the NIH Policy on the Inclusion of Women and Minorities as Participants in Research Involving Human Subjects. Regarding inclusion of women, female residents will make up approximately 26% of our sample. This is comparable to the natural proportion of females expected to be residents of SRHT. We expect that about 11% of the SRHT resident participants will be Hispanic and 70% will be African American (see Planned Enrollment Table).

Pregnant young women will not be excluded since no procedure in this study would pose a threat to the health and well-being of a pregnant woman or her fetus. However, we will not request or collect data on pregnancy at screening.

Skid Row Housing Trust (SRHT) planning. The SRHT will receive compensation for the staff time required for identifying case managers to be part of the study and residents for beta-testing and the pilot-test. Details for compensation for each year are provided in the Budget Justification section. Administrators, case manager supervisors and case managers will participate in planning activities. We do not treat participants in planning activities as human subjects because they will be speaking in their professional capacity.

Beta-testing and feasibility/acceptability interviews. We will conduct 8-12 beta test interviews with residents of SRHT. First, we will coordinate with SRHT to recruit 4-6 residents to review the MNI prototype. We will work with SRHT prior to recruitment to develop a script to be given to residents/read to residents during recruiting to explain the purpose of the study, the potential risks, the benefits of the study, and to inform them of their rights to refuse to participate in the study. The script will be developed with the approval of the RAND HSPC. RAND will conduct the initial 4-6 beta test interviews. Data from these interviews will be collected on the MNI prototype and the interviews will also be audio-recorded with the resident's consent. After each beta-test MNI, we will ask residents about their experiences (e.g., what your overall impressions of the session were; what was most helpful/unhelpful; what did you like/dislike/would like change). We will also walk through the MNI screens with residents for feedback and explore if the wording or visualizations of the screens are difficult to understand. In the second phase, we will work with SRHT administrators and supervisors to identify 8 case managers to participate in the second phase of beta-testing. We will develop a second script to inform case managers about the study and their rights as participants in the beta-test (if they are one of the 4 case managers selected at random to deliver the MNI) and pilot test and ask for oral consent. We will use the same script developed in phase 1 for a new set of 4-6 residents which will be recruited by SRHT. Dr. Osilla will train 4 case managers to deliver the MNI to 4-6 residents. RAND will then independently interview residents and case managers about their experience. The purpose of this phase of beta-testing is to further assess the residents' experience and to assess case manager's experience (e.g. ease of use of the tablet, navigation through screens, what errors are common, how easy is it to correct these errors) when interacting with the MNI tool. We will also assess perceived barriers to using the MNI during case management sessions to identify

potential barriers for Aim 2 of the study (e.g., what problems might we face if case managers had to use the MNI with several different residents? What are some ideas for solving those problems?). Residents will receive $25 for beta-testing and we will compensate SRHT for training and beta-testing time. One of the objectives of this planning is to identify the best procedures for contacting residents for the pilot-test and informing them of their rights as participants.

Potential participants will be recruited from initial intakes conducted by case managers at SRHT (Figure 2). The RAND research team will monitor the number of move-ins and intakes conducted to ensure that every adult resident is screened for eligibility and offered the opportunity to participate. Residents who meet study criteria and agree to become participants will be asked to complete baseline assessment interview and follow-up assessment interview *within 2 weeks after the final MNI session*. Residents will be paid $30 for the baseline assessment and $40 for follow-up. Eligible residents will be randomly assigned to MNI or usual case manager support. Those assigned to the MNI group will also be asked to complete a client satisfaction interview *during the follow-up assessment interview*.

Surveys of SRHT residents. Potential participants will be recruited from initial intakes conducted by case managers at SRHT (Figure 2). The RAND research team will monitor the number of move-ins and intakes conducted to ensure that every adult resident is screened for eligibility and offered the opportunity to participate. Those who are interested will be given information about their involvement in the intervention, the interviews they will be asked to complete, the potential risks, the potential benefits, and information informing them about their rights as participants, including informed consent. Residents who meet study criteria and agree to become participants will be asked to complete baseline assessment interview and post-intervention interview within two weeks after the final MNI session. Residents will be paid $30 for the baseline assessment and $40 for the post-intervention assessment. Eligible residents will be randomly assigned to MNI or usual case manager support. Those assigned to the MNI group will also be asked to complete a client satisfaction interview as part of their post- MNI interview.

Intervention. The SRHT program currently provides regular individualized case management meetings with each new resident. The proposed intervention is designed to specifically address the needs of new residents that would normally be covered in these supportive meetings, including discussions of their social networks.
In the screening for this intervention, residents will need to agree to be randomly assigned to receive the intervention MNI case manager meetings or usual care case management meetings. Residents will be informed that participation in the intervention is voluntary and they will not be compensated for the intervention sessions as they will be part of SRHT regular case management meetings with new residents.

As part of the MNI sessions, all meetings will be recorded, including usual care meetings, with residents' consent. Residents will be informed that the information that is discussed in the meetings will be confidential and that what is reported during the intervention sessions will not impact their service requirement for the SRHT.

During interventions, participants will be asked to discuss their network ties by first name, initials or nicknames only.

Sources of materials. Sources of research materials (data) include beta-test interviews of no longer than one hour in duration for residents and residents + case managers, two assessments at baseline and 3 months post-intervention of one hour in duration, and one post intervention satisfaction survey for residents randomized into the MNI. Assessments will not be audio recorded. Data will be collected electronically on laptops with computer assisted survey software and participants will be de-identified. In the baseline and 3 months post-intervention assessments, participants will be asked to identify and discuss network ties by first name, initials, or nicknames only. No existing data for individual residents or specimens will be used.

Potential risks. There are no physical risks to participants. Psychological and social risks are expected to be minimal (e.g., embarrassment/distress about answering questions related to substance involvement, risky sex) and these risks are no higher for intervention participants than under usual care conditions. Residents already discuss substance use and risky sexual behavior with case managers. One risk of the current project is breach of confidentiality. Although this would be very serious if it were to occur, breach of confidentiality is

highly unlikely as data are used exclusively for research purposes and residents will be provided with information on how risks to confidentiality are minimized (see protection against risks for further details).

Adequacy of protection against risks

Recruitment and informed consent

Beta-test interviews. Residents who participate in the beta-tests must consent to participate in the study when they are recruited by case managers. Those who receive consent will be scheduled to participate in a beta-test interview as described above, SRHT staff who participate in the beta-test and pilot-tests will be speaking only in their professional capacity, thus we do not treat them here as human subjects and plan to seek oral consent.

Survey. Informed consent will be obtained from interested residents prior to participation in the assessments. At one of the initial case management meetings with a new resident, a description of the study and consent procedures will be provided to all residents who screen eligible. They will be given a toll free phone line throughout the screening and data collection fieldwork periods. RAND staff (up to the level of the principal investigator) will be available to train SRHT staff and answer any questions about the study or consent procedures.

RAND staff will conduct the immediate posttest survey and the RAND survey staff will be responsible for surveying respondents who are no longer residents and at the 3-month follow-up assessment. To obtain residents' assent, all staff administering the surveys will be trained to read a short statement at the beginning of the survey session that explains the survey and what they will be asked to do. The consent for participation in the pre-intervention (baseline) assessment will include a request to be re-contacted to complete additional questionnaires. Consent will be requested at each data collection point. Those residents who agree to complete the questionnaire will sign an assent statement. They will also be asked to sign a participant locator/tracking form giving us permission to use the contact information they provide to locate them for future survey waves. Participation will be voluntary and a full informed consent protocol will be developed under the guidance of RAND's Human Subjects Protection Committee. Residents will be assured that participation is voluntary and that non-participation will not result in any consequences to them. In addition, they will be informed that they may withdraw from the study at any time without consequences. Finally, residents are informed that the data are used exclusively for research purposes and that identifying data will not be released by the investigators to SRHT or to any other individual or organization and that any identifying information is destroyed at the end of the project.

In soliciting the cooperation with SRHT in our study, we will stress the many data safeguards that will be taken to protect the confidentiality of the survey data. Minimizing the risk of disclosure requires careful data safeguards in the field and at RAND. Most important is the need to prevent the association of an individual resident's name with sensitive information. Following the procedures successfully implemented for our previous NIH funded projects, we will keep participants' names separate from their survey responses and store the link between names and survey IDs in a locked facility accessible only to authorized staff. In addition, to ensure that such information is protected from subpoena from law enforcement agencies, we will apply for a Confidentiality Certificate from the US Department of Health and Human Services.

Protection against risks. RAND is experienced at handling sensitive and confidential data. Certain routine administrative, personnel, physical security, information management, and computer system or network security practices are always in place given RAND's policies and the requirements for safeguards consistent with the management of Department of Defense classified data. Participants in the current study will be assigned an identification number, to be used on their pre-, post-, and follow-up intervention questionnaires. Thus, no assessment will contain identifying information, and the list that links identification numbers to names will be kept separately and locked. At the completion of data collection, this list will be destroyed. The data collectors will follow a strict written protocol that describes study measures for protecting data privacy, explains that each resident has the right to refuse to participate or to refuse to answer any individual question that they find objectionable. The RAND Human Subjects Protection Committee requires a rigorous data safeguarding procedure to protect confidentiality.

Data handling, software, and storage and disposition. Raw data will be cleaned and edited by RAND. Audio recordings will be uploaded from a recording device to a SRHT computer that is connected to the internet. RAND will provide login capabilities to its secure project management server (Teamspace) to one supervisor who will be responsible for uploading audio files so that they can be evaluated by Dr. Osilla. The audio recordings will be deleted immediately after confirmation that the files have been uploaded. RAND interviewers will use encrypted, password-protected laptops to collect assessment data and data files will be uploaded to the RAND server once interviewers are connected to the RAND network via Ethernet connections at the RAND Santa Monica office. Raw data will be cleaned and databases will not contain any identifying information. We will develop a master database for each wave of data collection for all participants. Data will be cleaned after each wave is collected, so that data analysis can begin immediately after the second wave of data is collected.

Minimizing the risk of disclosure requires careful data safeguards in the field and at RAND. Following the procedures successfully implemented for our other projects, we will keep participants' names separate from their survey responses and store the link between names and survey IDs in a locked facility accessible only to authorized staff. The RAND HSPC requires a rigorous data safeguarding procedure to protect confidentiality. We will work iteratively with SRHT to submit a detailed set of procedures for handling data to the RAND HSPC and will work with them to resolve any concerns about data handling to produce a final set of procedures.

Potential benefits of the proposed research to the subjects and others

We expect that the minimal risks faced by the residents in our study will be greatly outweighed by the benefits. Involvement in the study may help reduce substance use and HIV risk behavior among residents in this vulnerable population. Participants will learn detailed information about the functioning of their social networks. In other studies in which we have presented visualizations of social networks immediately after the interview, we found that participants generally are enthusiastic about seeing the networks and appreciate the opportunity to see their social lives in a visualized form.

The SRHT has already agreed to provide access to their residents because of the need for greater tools to provide assistance to them as they transition out of chronic homelessness to residence in permanent supportive housing. Staff involved in the study (i.e., beta-testing, recruitment, intervention delivery) will be compensated for their time and will receive valuable training in motivational interviewing techniques. SRHT will benefit by receiving information about the social network challenges facing their residents and this knowledge will ultimately contribute to a reduction in substance use and HIV risk among their residence.

Importance of the knowledge to be gained

This study should yield important new information contributing to prevention science and practice, particularly for Housing First programs and residents. The idea from the study comes in part from feedback received from homeless service organizations who participated in a NIH funded grant to study the social networks of heterosexual homeless men in Los Angeles. The development of a feasible and effective social network intervention based in motivational interviewing for Housing First residents should far outweigh any risk of harm.

Collaborating Sites

We will collaborate with the SRHT operated in the Skid Row area of Los Angeles, CA. Shannon Parker, Manager of Resident Services, will serve as our SRHT contact person and will primarily interface with Dr. Hunter.

Women, Minority and Children Inclusion in Clinical Research

A. Inclusion of women. Female residents are included in the sample at rates that are comparable to the naturally occurring population in the SRHT residential program (26%). Stratified sampling will assure that females are represented with similar proportions in each arm of the study.

B. Inclusion of Minorities. Racial and ethnic minorities are included in the sample at rates comparable to the naturally occurring population in the SRHT program.

C. Inclusion of Children. There will be no recruitment of any children as part of the proposed study.

Data and Safety Monitoring Plan

We target our Data Safety and Monitoring Plan to residents of Skid Row Housing Trust. This study does not involve any intervention posing a risk to the lives or physical health of the participants. In the beta-test phase of the study, interviews will be conducted by either Drs. Kennedy, Osilla, or Hunter who will rely on extensive note taking and listening to the audio files to record feedback. No names will be associated with the feedback, only the ideas or concepts that the residents and case managers conveyed during the beta-test interviews will be recorded. A manual will be developed for intervention implementation and adherence to the procedures will be monitored.

During intervention delivery, SRHT case managers will be trained as interventionists and will have weekly contact with Dr. Osilla, who is a licensed clinical psychologist in the state of California and is affiliated with the Motivational Interviewing Network of Trainers. They will discuss the on-going MNI deliveries, any fidelity issues that Dr. Osilla has identified, and any clinical issues that have occurred since the previous time they spoke. If an adverse event occurs (see below), Drs. Osilla and Kennedy will be notified immediately.

For assessments, questionnaire data will be collected at three different time points, at baseline, immediate post-test, and a three-month follow up. Drs. Kennedy and Hunter will be responsible for monitoring the safety and efficacy of this study, implementing the Data and Safety Monitoring Plan (DSMP), and complying with the reporting requirements. A summary of the DSMP report will be sent to both the Human Subjects Protection Committee (HSPC) at RAND and NIDA as part of the annual progress reports. The DSMP report will include information on participants; demographic characteristics, recruitment, treatment, retention, Adverse Events (AE's), Serious Adverse Events (SAE's), and any significant changes to the research procedures that affect the safety of human subjects.

Data Monitoring

<u>Data handling and quality.</u> Questionnaires will be administered to residents who participate in the study at three different time points. All aspects of data collection and data storage will be carefully monitored by Drs. Kennedy and Golinelli to ensure rapid detection of errors, inconsistencies or other problems. The data collectors will follow a strict written protocol that describes study measures for protecting data privacy, explains to each participant that s/he has the right to refuse to participate or to refuse to answer any individual question that s/he finds objectionable, and emphasizes the importance of telling the truth. RAND is experienced at training data collection fieldwork personnel in handling sensitive and confidential data and in the handling, storage and processing of confidential data. Certain routine administrative, personnel, physical security, information management, and computer system or network security practices are always in place given RAND's policies and the requirements for safeguards consistent with the management of Department of Defense classified data. These practices include building and network security, non-disclosure pledges, and account/password/encryption security on computer networks. In addition, we take multiple project-specific steps to protect subjects from the risk of a breach in confidentiality. All project staff sign confidentiality agreements requiring them to keep such information private. Second, all data will be collected using coded ID numbers. Thus, no questionnaire will contain identifying information, and the list that links identification numbers to names will be kept separately in a locked file. This key will be maintained until follow-up questionnaires have been completed, at which time the key will be destroyed. Third, we will apply to DHHS for a Certificate of Confidentiality protecting collected data from subpoena. Finally, only aggregate data that cannot be used to identify individuals will be included in any reports released to other agencies or for publication.

Safety Monitoring Plan

<u>Population.</u> The beta-testing, assessment interviews, and intervention deliveries will involve the vulnerable population of formerly chronic homeless adult residents of a permanent supportive housing program. These residents are monitored by the staff at SRHT where they reside and receive a wide range of services individually tailored to their needs. It is unlikely that participation in beta-testing, secondary intervention or assessments will cause residents to experience an "adverse event", however, trained staff will be conducting both the beta-test interviews and the intervention sessions to ensure that residents are stable enough to participate in this study and to address any potential crises. In addition, Drs. Osilla and Hunter will be supervising intervention implementation and fidelity throughout the study.

Monitoring. Drs. Kennedy, Osilla, and Hunter will conduct the beta-test interviews. Dr. Kennedy has extensive experience designing and conducting qualitative interviews with various populations about sensitive subjects and has extensive experience with qualitative interviews with homeless populations. Dr. Hunter also has extensive experience with the population of residents of SRHT and Dr. Osilla has extensive experience interacting with substance use intervention subjects. Should any adverse events occur during the interviews, we will use standard HSPC and NIH procedures for reporting these events. Concerning the implementation of the intervention, residents of SRHT are supported by the SRHT staff. All case managers will receive weekly supervision by Dr. Osilla.

Adverse Events. For purposes of monitoring and reporting adverse events, the following NIH definitions will be used:

Adverse Event (AE): any untoward medical occurrence that may present itself during participation or administration of an intervention, and which may or may not have a causal relationship with the participation.

Serious Adverse Event (SAE): Any medical occurrence that results in death; is life-threatening; requires inpatient hospitalization or prolongation of existing hospitalizations; creates persistent or significant disability/incapacity, or a congenital anomaly/birth defect. Such an event could include suicidal ideation or attempted drug overdoes or withdrawal, etc.

Dr. Osilla will be providing weekly supervision to the SRHT case managers and will be listening to the audio recordings of the MNI deliveries each week. It is highly unlikely that participation in case manager MNI meetings will cause an AE or SAE. Should an AE occur, however, the case manager will report any AE's connected to the implementation of the intervention immediately to Drs. Osilla and Kennedy and they will keep a log of AE's and SAE's. Dr. Kennedy has managed AEs in a research capacity as the primary contact person for qualitative and social network interviews with depression intervention recipients and has facilitated communication with clinical contacts for AEs and SAEs. He will serve the same role on this project. Drs. Kennedy, Osilla and Hunter will determine if an SAE is related to the research project and will file an immediate written report to the HSPC and NIDA Project Officer, whether the event relates to the project or not. The log will be used to provide information about adverse events in annual progress reports to the HSPC and NIDA. Outcomes for SAE's will be monitored by Drs. Kennedy, Osilla and Hunter, and outcome information will be entered into a log for inclusion in reports to the HSPC and NIDA.

Should any other problems or other concerns arise with the survey or intervention program, the PI and Co-PI will be on call to address these with the staff that are implementing the survey (e.g., if a resident is distressed about answering sensitive questions on the survey). In addition, resource and referral listings for community mental health services will also be provided on a routine basis as needed. Drs. Kennedy, Osilla and Hunter will also work extensively with the staff at SRHT to ensure that if participants need to be referred that these situations are managed in a manner consistent with probation preferences/policies

INCLUSION OF WOMEN AND MINORITIES

Inclusion of Women. This study includes women and complies with *NIH Policy on the Inclusion of Women and Minorities.* We estimate that 26% of our sample will be women, which is representative of the gender distribution of Skid Row Housing Trust residents.

Inclusion of Minorities. This study includes ethnic minorities who are residents of Skid Row Housing Trust's residential housing program and complies with *NIH Policy on the Inclusion of Women and Minorities.* Based on a 2010 demographic report produced by Skid Row Housing Trust, we estimate that the residents we sample will be primarily Black/African-American (73%), Hispanic/Latino (11%) or White (8%). We expect that Native Americans, Asians and Hawaiian/Pacific Islanders will make up less than 8% of the sample.

No specific outreach programs will be used to recruit ethnic minorities as they are well represented in the population of Skid Row Housing Trust residential facilities. We will stratify our screening procedures by gender to assure that we have equal representation of men and women across the two arms of the study.

Targeted/Planned Enrollment Table

This report format should NOT be used for data collection from study participants.

Study Title: Enhancing Housing First Programs with a Social Network Substance Use Intervention

Total Planned Enrollment: 72

TARGETED/PLANNED ENROLLMENT: Number of Subjects			
**Ethnic Category**	**Sex/Gender**		
	**Females**	**Males**	**Total**
Hispanic or Latino	2	6	8
Not Hispanic or Latino	17	47	64
**Ethnic Category: Total of All Subjects ***	19	53	72
**Racial Categories**			
American Indian/Alaska Native	0	1	1
Asian	0	1	1
Native Hawaiian or Other Pacific Islander	0	0	0
Black or African American	14	39	53
White	2	4	6
**Racial Categories: Total of All Subjects ***	19	53	72

* The “Ethnic Category: Total of All Subjects” must be equal to the “Racial Categories: Total of All Subjects.”

INCLUSION OF CHILDREN

We will recruit residents and staff (Administrators and Case Mangers) from Skid Row Housing Trust. We will not enroll anyone under the age of 18 in our study and do not expect to recruit any staff under the age of 21 given that the credentials for the job duties of the individuals we will be targeting require advanced training.
